# Supplementary material for: Feasibility of comparing medical management and surgery (with neurosurgery or stereotactic radiosurgery) with medical management alone in people with symptomatic brain cavernoma – protocol for the Cavernomas: A Randomised Effectiveness (CARE) pilot trial
Source: BMJ Open. 2023 Aug 9;13(8):e075187. doi: 10.1136/bmjopen-2023-075187 (PMC10414059; doi:10.1136/bmjopen-2023-075187)
Supplement: Supplementary data [file bmjopen-2023-075187supp002.pdf]

CARE Trial Steering Committee Charter

**Study Title:**	Cavernomas A Randomised Effectiveness (CARE) pilot trial, to address the effectiveness of active treatment (with neurosurgery or stereotactic radiosurgery) versus conservative management in people with symptomatic brain cavernoma
**Funder and funder reference:**	National Institute for Health Research Health Technology Assessment Programme - NIHR128694
**Chief Investigator:**	Prof Rustam Al-Shahi Salman
**Co-Sponsors:**	University of Edinburgh & NHS Lothian
**Sponsor reference:**	AC20171
**Trial Registration Reference(s):**	ISRCTN41647111
**REC reference:**	21/YH/0046
**Charter Version Number and Date:**	V3.0 (08 Mar 2023)<br>*Based on Sponsor Template CR015-T02 v2.0*

Loan JJM, *et al. BMJ Open* 2023; 13:e075187. doi: 10.1136/bmjopen-2023-075187

accord
Academic and Clinical Central Office for Research and Development

THE UNIVERSITY of EDINBURGH

NHS Lothian

Approval Signatures:

The following individuals, by providing their signatures, indicate their understanding of and willingness to comply with the roles and responsibilities assigned to them in this Charter.

1. TSC Independent Chair:

   Prof Garth Cruickshank

   ________________ ________________ __/__/____

   PRINT NAME SIGNATURE DATE

2. Prepared by Trial Manager:

   Dr Laura Forsyth

   ________________ ________________ __/__/____

   PRINT NAME SIGNATURE DATE

3. Chief Investigator:

   Prof Rustam Al-Shahi Salman

   ________________ ________________ __/__/____

   PRINT NAME SIGNATURE DATE

Loan JJM, *et al. BMJ Open* 2023; 13:e075187. doi: 10.1136/bmjopen-2023-075187

Table of Contents

1 Introduction 4
2 Roles and Responsibilities 4
3 Before or early in the trial 5
4 Composition 6
5 Relationships 8
6 Organisation of TSC Meetings 8
7 Trial Documentation and Procedures to Ensure Confidentiality and Proper Communication 9
8 Decision Making 10
9 Reporting 11
10 After the Trial 11
Appendix 1: Agreement and competing interests form for independent members .. 12
Appendix 2: Agreement and competing interests form for non-independent members 13
Appendix 3: Agreement and confidentiality agreement for observers 14

Loan JJM, *et al. BMJ Open* 2023; 13:e075187. doi: 10.1136/bmjopen-2023-075187

1 Introduction

This Charter is for the Trial Steering Committee (TSC) for the Cavernomas A Randomised Effectiveness (CARE) pilot trial, a pilot randomised controlled trial (RCT) which aims to assess the feasibility of conducting a definitive main phase RCT to address the research question "How effective is active treatment (with neurosurgery or stereotactic radiosurgery) versus conservative management in people with symptomatic brain cavernoma?". The trial objectives are to:

1. Engage a collaboration of specialists and patient advocacy groups in the UK and Ireland.
2. Establish a pilot RCT, with an embedded qualitative study to understand the anticipated recruitment processes and address any barriers.
3. Assess the feasibility of performing a definitive main phase of the RCT.

This charter will define the primary responsibilities of the TSC, its membership, and the purpose and timing of its meetings. It will also provide the procedures for ensuring confidentiality and proper communication, decision making, reporting and after trial publications. The trial will be conducted in accordance with sponsor SOPs (https://www.accord.scot/research-access/resources-researchers/sop). The contents of the Charter are based on the NIHR Research Governance Guidelines for Trial Steering Committees (https://www.nihr.ac.uk/documents/research-governance-guidelines/12154).

2 Roles and Responsibilities

The role of the TSC is to provide overall supervision for this project on behalf of the Project Sponsor (ACCORD) and Project Funder (NIHR HTA) and to ensure that the project is conducted to the rigorous standards set out in the Department of Health's Research Governance Framework for Health and Social Care and the Guidelines for Good Clinical Practice.

The specific roles of the TSC include:

- Provide oversight of the trial and monitor the overall conduct of the trial. The TSC should provide advice through its independent Chair to the Chief Investigator (CI) and Trial Management Group (TMG) on all appropriate aspects of the trial.
- Concentrate on progress of the trial, QuinteT Recruitment Intervention (QRI) progress and recommendations, adherence to the protocol, patient safety and the consideration of new information of relevance to the research question
- Ensure appropriate ethical and other approvals are obtained in line with the project plan
- Review regular trial progress reports
- Monitor recruitment rates and advise the TMG about strategies to deal with recruitment issues

- Monitor follow-up completeness and advise the TMG about strategies to deal with retention issues
- Review serious adverse events blind to treatment allocation
- Assess the impact and relevance of any accumulating external evidence (any relevant external evidence identified by the CI will be passed onto the TSC Chair for review by the committee)
- Review and accept/reject recommendations from the DMC to amend the protocol or conduct of the study
- Contribute to enhancing the integrity of the trial. The TSC may also formulate recommendations relating to:
  - The selection, recruitment, or retention of participants, or their management
  - Extending recruitment or follow up
  - Improving participant adherence to protocol-specified regimens
  - Procedures for data management and quality control
- Promptly review DMC recommendations which include deciding to continue or terminate the trial
- Oversee the timely reporting of the trial results
- Maintain confidentiality of all trial information that is not already in the public domain
- Comment on the main trial manuscript before publication (if desired)

3 Before or early in the trial

All potential TSC members will have sight of the protocol before the first TSC meeting. Before recruitment begins, the trial will have undergone review by the sponsor and a research ethics committee. Therefore, if a potential TSC member has major reservations about the trial (e.g. the protocol or the logistics) they should report these to the CI and may decide to decline the invitation to join. TSC members should be constructively critical of the ongoing trial, but also supportive of aims and methods of the trial.

The TSC will meet before the start of recruitment to the trial, to discuss the protocol, methods of providing information to and from the TSC, frequency and format of meetings, relationships with other committees and have the opportunity to clarify any aspects with the CI and Co-Chief Investigator. TSC input into the protocol will be discussed with the CI before deciding what protocol updates need to be implemented.

Members and observers of the TSC will not be asked to formally sign a contract but should formally register their assent by confirming (1) that they agree to be a member of the TSC and (2) that they agree with the contents of this Charter by signing and dating the required form (Appendices 1-3).

Loan JJM, et al. BMJ Open 2023; 13:e075187. doi: 10.1136/bmjopen-2023-075187

4 Composition

TSC members were selected and approved by the funder in accordance with NIHR Research Governance Guidelines (V1.0 February 2019).

The Chairperson

The Chair of the TSC will be independent of the trial and have experience of serving on previous TSC(s). The Chair is directly answerable to the relevant NIHR programme, as funder and the primary TSC reporting line is via the Chair to the relevant NIHR Programme Director; however communication is likely to be between the Chair, the trial manager and the NIHR Research Manager who has day to day responsibility for the project.

The Chair's specific responsibilities include:

- Liaising with the CI to arrange a meeting to finalise the protocol and to set up a schedule of meetings to align with the project plan
- Establishing clear reporting lines to the Funder, Sponsor, etc.
- Being familiar with relevant guidance documents and with the role of the DMC, if appropriate
- Providing an independent*, experienced opinion if conflicts arise between the needs of the research team, the funder, the sponsor, the participating organisations and/or any other agencies
- Leading the TSC to provide regular, impartial oversight of the study, especially to identify and pre-empt problems
- Ensuring that changes to the protocol are debated and endorsed by the TSC. Letters of endorsement should be made available to the project team when requesting approval from the funder and sponsor for matters such as changes to protocol
- Being available to provide independent* advice as required, not just when TSC meetings are scheduled
- Commenting on any extension requests and, where appropriate, providing a letter to the funder commenting on whether the extension request is supported or otherwise by the independent* members of the TSC
- Commenting in detail (when appropriate) regarding the continuation, extension or termination of the project. NB: The TSC Chair does not need to be a content expert him/herself but needs to ensure that sufficient content expertise is available for the group to perform its oversight function effectively

*** Independence**

According to the NIHR Research Governance Guidelines, independence is defined as:

- Not part of the same institution as any of the applicants or members of the project team
- Not part of the same institution that is acting as a recruitment or investigative centre, including Patient Identification Centres (PIC), identifying and referring

patients to a recruitment or investigative centre (in both cases, 'not part of the same institution' means holding neither a substantive or honorary contract with said institution)

- Not related to any of the applicants or members of the project team
- For the chair only: not an applicant on a rival proposal

TSC membership and voting

The TSC will consist of a minimum of 75% independent members. Only appointed TSC members will be entitled to a vote and the chair will have a casting vote. To minimise the risk that fewer than 75% of TSC members are independent at a TSC meeting, the CI is an observer and not formally a member of the TSC for this trial and therefore cannot vote. Attendance of non-members at meetings is at the discretion of the TSC Chair.

The **members** of the TSC are listed below.

Name of Member	Role in TSC	Responsibility	Independent
Prof Garth Cruickshank	Independent Chair	Provide independent neurosurgical and trial expertise	Y
Prof Catherine Hewitt	Independent member	Provide independent statistical expertise	Y
Mr Richard Kerr	Independent member	Provide independent vascular neurosurgery and trial expertise	Y
Prof Haleema Shakur-Still	Independent member	Provide independent clinical trial management expertise	Y
Mr Ian Stuart	Independent member	Patient/carer representative	Y
Mr David White	Independent member	Patient/carer representative	Y
Mr Neil Kitchen	Co-chief investigator	Neurosurgical lead	N

The **observers** of the TSC are listed below.

Prof Rustam Al-Shahi Salman	Chief Investigator	Inform TSC of any relevant updates	N
Prof Steff Lewis	Study Statistician	Blinded trial statistician	N
Dr Laura Forsyth	Trial Manager / Facilitator	Co-ordinate meetings and facilitate the group	N
Dr Julia Wade	Lead Qualitative Researcher	Report on the progress, conduct, and outcomes of the embedded QuniteT Recruitment Intervention	N

Loan JJM, et al. BMJ Open 2023; 13:e075187. doi: 10.1136/bmjopen-2023-075187

5 Relationships

TSC / DMC relationship

The TSC is the oversight body of the trial. All substantial issues regarding the trial must go to the TSC for consideration. The DMC is advisory to the TSC.

Payments to TSC members

If required, standard travel and accommodation costs will be paid to members of the TSC. No other payments or rewards will be given.

Competing Interests

Any competing interests, either real or potential, should be disclosed before TSC meetings (see Appendices). These are not restricted to financial matters, involvement in other trials or intellectual investment could be relevant. Although members may well be able to act objectively despite such connections, complete disclosure enhances credibility.

6 Organisation of TSC Meetings

Meeting Frequency and Format

The TSC should have a formal meeting at least yearly. At the request of the TSC, interim meetings will be organised. Meetings will be scheduled to follow shortly after DMC meetings so that any DMC recommendations can be considered, if appropriate. The responsibility for calling and organising TSC meetings lies with the CI who will be assisted by the Trial Manager/Facilitator.

Meetings will be held either in person, by video-conference (e.g. Zoom, MS Teams) or by teleconference. Major trial issues may need to be dealt with between meetings, by phone, video-conference or by email. TSC members should be prepared for such instances. There may be occasions when the Sponsor or the Funder will wish to organise and administer these meetings for particular projects. This is unlikely, but the NIHR reserves the right to attend any meeting therefore should be included in relevant invitations and also reserves the right to convene a meeting of the TSC in exceptional circumstances.

Attendance

Presence will be usually limited to the TSC members, observers and the Facilitator (and/or their delegate) however, other attendees such as representatives of the Funder and Sponsor may also be invited to all or part of every meeting by the TSC. Other observers who are not members of the TSC may be invited to provide expert input.

Effort will be made to ensure that all members can attend. The CI must try to attend all meetings, especially if major actions are expected. In the case of face to face meetings, members who cannot attend in person will be encouraged to participate by teleconference/videoconference. If TSC members cannot attend meetings by tele-/video-conference, they will be encouraged to send comments in advance via email.

Loan JJM, *et al. BMJ Open* 2023; 13:e075187. doi: 10.1136/bmjopen-2023-075187

Quoracy

If, at short notice, any TSC members cannot attend then the TSC may still meet if at least five members (two thirds of the appointed membership) including the Chair will be present, plus a member of the trial team. If the TSC is considering a major action after such a meeting the TSC Chair should communicate with the absent members, including the CI, as soon after the meeting as possible to check they agree. If they do not, a further meeting should be arranged with the full TSC.

Non-attendance

TSC members who will not be able to attend the meeting should pass comments to the TSC Chair in advance for consideration during the discussion. If an independent member does not attend a meeting or provide comments when requested between meetings, it will be ensured that the independent member is available for the next meeting. If an independent member does not attend the next meeting or provide comments when next requested, they will be asked if they wish to remain part of the TSC. If an independent member does not attend a third meeting, strong consideration will be given to replacing this member.

7 Trial Documentation and Procedures to Ensure Confidentiality and Proper Communication

Progress Report and Meeting Minutes

At the first meeting, the TSC will review the project plan and discuss targets for recruitment, data collection, compliance etc. Based on these targets, the TSC should agree a set of data that should be presented in a progress report at each meeting. The progress report will be written and presented by the Chief Investigator (or designee) and will include updates on trial progress, recruitment, participant drop-out, safety data (SAEs), adherence to the protocol (deviations and violations), summary of new evidence/literature review, publications and A.O.B, as appropriate. The TSC will receive the report and any associated documentation at least two weeks before the meeting.

Minutes will be prepared by the facilitator on behalf of the CI, and uploaded to the NIHR MIS. Copies of minutes will be sent to all members, the sponsor and the funder, and a copy will be retained in the Trial Master File. These minutes and actions will be used as a basis for the following TSC meeting agenda.

External evidence

Identification and circulation of published external evidence (e.g. from other trials/ systematic reviews) is a responsibility of the CI. The TSC should continue to be made aware of other data that may impact on the trial.

Communication

The facilitator will be responsible for the organisation of meetings and should be copied into all communications with and between the TSC.

Confidentiality

TSC members are expected to store securely copies of the reports to and from the

TSC, agenda and minutes, as well as copies of communications between meetings. All documentation should be considered confidential.

8 Decision Making

TSC / DMC decision-making

The TSC is jointly responsible with the DMC for safeguarding the interests of participating patients and for the conduct of the trial. Recommendations to amend the protocol or conduct of the study made by the DMC will be considered and accepted or rejected by the TSC. The TSC will be responsible for deciding whether to continue or to stop the trial based on the DMC recommendations.

Possible decisions by the TSC include:

- No action needed, trial continues as planned
- Early termination of the trial (e.g. because of harm of treatment or futility or external evidence. This would generally be after a recommendation from the DMC).
- Stopping recruitment within a subgroup
- Extending recruitment or extending follow-up
- Sanctioning or proposing protocol changes

Based on other factors, other possible decisions could include:

- Approving proposed new trial sub-studies
- Approving presentation of results during the trial or soon after closure
- Approval of strategies to improve recruitment or follow-up
- Approving feasibility of proceeding to a definitive main phase trial application

Considerations on statistical methods

Formal statistical methods may have been considered by the DMC in making their recommendations to the TSC. These methods are usually used as guidelines rather than absolute rules. This is because they generally only consider one dimension of the trial. The DMC will record reasons for disregarding stopping guidelines and will review and agree any interim analysis plan and note these decisions in their meetings and may choose to also note this in their report to the TSC if necessary.

Consensus and quoracy

Every effort should be made to achieve consensus. The role of the Chair is to summarise discussions and encourage consensus; therefore, it is usually best for the Chair to give their own opinion last. If a vote is required to achieve consensus, all independent members of the TSC have the opportunity to cast a vote with the chair voting last. The CI is not able to cast a vote.

To be quorate, at least five members (two thirds of the appointed membership) including the Chair will be present, plus a member of the trial team. It is important that the implications (e.g. ethical, statistical, practical, and financial) for the trial be considered before any decision is made.

The DMC will be notified of all changes to the protocol or to study conduct. The DMC's approval will be sought on all substantive recommendations or changes to the protocol or study conduct before their implementation.

9 Reporting

TSC recommendations

Notes of key points, decisions and actions will be made by the Facilitator. This will include details of whether potential competing interests have changed for any attendees since the previous meeting. The draft minutes will be initially circulated for comment to those TSC members who were present at the meeting. The TSC Chair will approve the final version of minutes within three weeks of the meeting and a copy sent to all attendees and the NIHR. Copies will be retained in the Trial Master File and archived at the time of study closure. The TSC may also provide feedback to the DMC, and where appropriate the Sponsor. Copies of communications will pass through the Facilitator.

The TSC is the oversight body for the trial. However the TSC should have good reason before deciding not to accept requests from the TMG or DMC. If there are serious problems or concerns with the TSC decision following a DMC recommendation, a joint meeting of the TSC and DMC should be held. The information to be shown would depend upon the action proposed and each committee's concerns. Depending on the reason for the disagreement confidential data and/or data by trial and may have to be revealed to all or some of those attending such a meeting: this would be minimised where possible. The meeting would be chaired by an external expert who is not directly involved with the trial.

10 After the Trial

Publication of results

The TSC will oversee the timely analysis, writing up and publication of the main trial results. The independent members of the TSC will have the opportunity to read and comment on the proposed main publications of trial data prior to submission and abstracts and presentations during the trial. This review may be concurrent to that of the trial investigators and DMC. TSC members will be named and their affiliations listed in the main report, unless they explicitly request otherwise.

Confidentiality of results

Unless permission has been agreed with the TSC, individual members will not discuss confidential information to which they have become party as a result of their involvement in the trial until 12 months after the primary trial results have been published.

Loan JJM, et al. BMJ Open 2023; 13:e075187. doi: 10.1136/bmjopen-2023-075187

Appendix 1: Agreement and competing interests form for independent members

Trial Steering Committee: Agreement to join the CARE Trial Steering Committee as an independent member and disclosure of potential competing interests

Please complete the following document and return to the TSC Facilitator.

(please **initial** box to agree)

☐	I have read and understood the CARE pilot trial TSC Charter version 3.0 dated 08 March 2023 and agree with the contents of this Charter
☐	I agree to join the Trial Steering Committee for this trial as an **independent** member
☐	I agree to treat all sensitive trial data and discussions confidentially

The avoidance of any perception that independent members of a TSC may be biased in some fashion is important for the credibility of the decisions made by the TSC and for the integrity of the trial.
Potential competing interests should be disclosed via the study office. In many cases simple disclosure up front should be sufficient. Otherwise, the (potential) independent TSC member should remove the conflict or stop participating in the TSC. **Table 1** lists potential competing interests.

☐	**No,** I have no competing interests to declare
☐	**Yes,** I have competing interests to declare (please detail below)

Please provide details of any competing interests:

NAME: ____________________

SIGNATURE: ____________________

DATE: ____________________

Table 1: Potential competing interests for independent members

- Stock ownership in any commercial companies involved
- Stock transaction in any commercial company involved (if previously holding stock)
- Consulting arrangements with the Sponsor/Funder
- Frequent speaking engagements on behalf of the intervention
- Career tied up in a product or technique assessed by trial
- Hands-on participation in the trial
- Involvement in the running of the trial
- Emotional involvement in the trial
- Intellectual conflict e.g. strong prior belief in the trial's experimental arm
- Involvement in regulatory issues relevant to the trial procedures
- Involvement in the writing up of the main trial results in the form of authorship

Loan JJM, *et al. BMJ Open* 2023; 13:e075187. doi: 10.1136/bmjopen-2023-075187

Appendix 2: Agreement and competing interests form for non-independent members

Trial Steering Committee: Agreement to join the CARE Trial Steering Committee as a non-independent member and disclosure of potential competing interests

Please complete the following document and return to the TSC Facilitator.

(please **initial** box to agree)

	I have read and understood the CARE pilot trial TSC Charter version 3.0 dated 08 March 2023 and agree with the contents of this Charter
	I agree to join the Trial Steering Committee for this trial as a **non-independent** member
	I agree to treat all sensitive trial data and discussions confidentially

The notion that non-independent members can act objectively despite potential competing interests is important for the credibility of the decisions made by the TSC and for the integrity of the trial. Potential competing interests should be disclosed via the study office. In many cases simple disclosure up front should be sufficient. Otherwise, the (potential) non-independent TSC member should remove the conflict or stop participating in the TSC. **Table 1** lists potential competing interests.

	**No,** I have no competing interests to declare
	**Yes,** I have competing interests to declare (please detail below)

Please provide details of any competing interests:

NAME: ____________________

SIGNATURE: ____________________

DATE: ____________________

Table 1: Potential competing interests for non-independent members

- Stock ownership in any commercial companies involved
- Stock transaction in any commercial company involved (if previously holding stock)
- Consulting arrangements with the Sponsor/Funder
- Frequent speaking engagements on behalf of the intervention
- Intellectual conflict e.g. strong prior belief in the trial's experimental arm
- Involvement in regulatory issues relevant to the trial procedures

Loan JJM, *et al. BMJ Open* 2023; 13:e075187. doi: 10.1136/bmjopen-2023-075187

Appendix 3: Agreement and confidentiality agreement for observers

Trial Steering Committee: Agreement to attend the CARE Trial Steering Committee and treat all information confidentially

Please complete the following document and return to the TSC Facilitator.

(please **initial** box to agree)

	I agree to attend the Trial Steering Committee meeting on ___/____/______
	I agree to treat as confidential any sensitive information gained during this meeting and all future meetings unless explicitly permitted

NAME: ______________________________

SIGNATURE: ______________________________

DATE: ______________________________

Loan JJM, *et al. BMJ Open* 2023; 13:e075187. doi: 10.1136/bmjopen-2023-075187
