# Supplementary material for: Feasibility of comparing medical management and surgery (with neurosurgery or stereotactic radiosurgery) with medical management alone in people with symptomatic brain cavernoma – protocol for the Cavernomas: A Randomised Effectiveness (CARE) pilot trial
Source: BMJ Open. 2023 Aug 9;13(8):e075187. doi: 10.1136/bmjopen-2023-075187 (PMC10414059; doi:10.1136/bmjopen-2023-075187)
Supplement: Supplementary data [file bmjopen-2023-075187supp003.zip › 02 PIL & CF/CARE - Record of Consultation Adult Participant Representative V1.0 (27Jan2021).docx]

**CARE pilot trial (Randomised Study)**

**Record of Adult Participant Representative Consultation on Continued Participation (Loss of Mental Capacity in Adult Participant)**

**Please (1) Refer to the original Informed Consent Form signed by the participant, and then (2) complete Part A OR Part B of this form**

***Part A - To be completed where the Participant Representative does not wish to amend the consent previously provided by the participant***

Relationship to participant ________________________________

I confirm that I am the representative for participant ­__________________________ and that no other nearest relative or welfare attorney or welfare guardian exists (Scotland and Republic of Ireland).

Or

I confirm that I am the consultee for participant ­__________________________ (England/ Wales/ Northern Ireland).

| Please **initial** box | |
| --- | --- |
| 1. I confirm that I have been consulted about the participant’s continuing participation in the above project. I have read and understand the patient information sheet (Version Number:___, Date:____________) for the CARE pilot trial. I have had the opportunity to consider the information, ask questions and have had these questions answered satisfactorily. |  |
| 1. In my opinion, the participant would have no objection to continuing to take part in the above study. |  |
| 1. I understand that I am free to withdraw the participant at any time, while they lack mental capacity, without giving a reason and without their medical care and/or legal rights being affected. |  |

|  |  |  |  |  |
| --- | --- | --- | --- | --- |
| Name of Participant Representative |  | Date |  | Signature |
|  |  |  |  |  |
| Name of Person Undertaking Consultation (researcher) |  | Date |  | Signature |

| ***Part B - To be completed where the Participant Representative requests changes to the consent previously provided by the participant***  Relationship to participant _______________________­­­­­­­­­____________  I confirm that I am the representative for participant ­__________________________ and that no other nearest relative or welfare attorney or welfare guardian exists (Scotland and Republic of Ireland).  Or  I confirm that I am the consultee for participant ­__________________________ (England/ Wales/ Northern Ireland).  Please **initial** box | |
| --- | --- |
| 1. I confirm that I have been consulted about the participant’s continuing participation in the above project. I confirm that I have read and understand the patient information sheet (Version Number:___, Date:____________) for the CARE pilot trial. I have had the opportunity to consider the information, ask questions and have had these questions answered satisfactorily. |  |
| 1. In my opinion, the participant would wish for the following changes to be made to his/her consent previously provided. |  |
| 1. I understand that the participant’s participation is voluntary and that I am free to withdraw them at any time, without giving any reason and without their medical care and/or legal rights being affected. |  |
| 1. I give permission for the research team to continue to access the participant’s medical records for the purposes of this research study. |  |
| 1. I understand that relevant sections of their medical notes and data collected during the study may be looked at by individuals from the Sponsor (University of Edinburgh and/or NHS Lothian), from regulatory authorities or from the NHS organisation where it is relevant to them taking part in this research. I give permission for these individuals to have access to the participant’s data and/or medical records. |  |
| 1. I give permission for my personal information and that of the participant I represent (including name, address, email address, date of birth and telephone number) to be entered into an electronic database managed by the Edinburgh Clinical Trials Unit and that relevant staff will have access to this information for administration purposes. I understand that my contact details will be used to contact me about the study. |  |
| 1. I give permission for their NHS number, Community Health Index (CHI) number or equivalent in other nations, and hospital number to be collected and passed to the Edinburgh Clinical Trials Unit. |  |
| 1. I agree to the participant’s General Practitioner being informed of their participation in the study and providing follow-up information about their current and future health problems for the lifetime of the study. |  |
| 1. I understand that data collected about the participant during the study may be converted to de-identified data. |  |
| 1. I agree that the researchers can find out how the participant is getting on by contacting me every 6 months for the duration of the study follow up period. |  |
| 1. The participant’s relatives, carers, or close personal contacts named on the Contact Form are willing to provide information about how the participant is getting on. These people or others caring for them may provide this information if I cannot be contacted |  |
| 1. I agree to the participant giving a blood sample which will be used for genetic DNA analysis. | Yes No |
| 1. I agree to the following being shared with other researchers and being used in future studies, whatever happens to the participant:  - De-identified data - De-identified brain imaging - De-identified blood or DNA sample | Yes No  Yes No  Yes No |
| 1. I understand that the information held and maintained by NHS Digital and other central UK NHS bodies (or the equivalent in other nations) may be used to help contact the participant or provide information about their health and use of services. I agree to their NHS, Community Health Index (CHI) number or equivalent in other nations being used to gather information relevant to their participation after the study has finished. | Yes No |
| 1. The participant should be informed of the results of the CARE pilot trial. | Yes No |
| 1. I agree to the participant’s contact details being kept on record and that they are happy to be contacted about similar research in the future. | Yes No |
| 1. I agree to the participant continuing to take part in this study. |  |

|  |  |  |  |  |
| --- | --- | --- | --- | --- |
| Name of Participant Representative |  | Date |  | Signature |
|  |  |  |  |  |
| Name of Person Undertaking Consultation (researcher) |  | Date |  | Signature |

1x original – into Site File; 1x copy – to Participant; 1x copy – into medical records
