# Supplementary material for: Feasibility of comparing medical management and surgery (with neurosurgery or stereotactic radiosurgery) with medical management alone in people with symptomatic brain cavernoma – protocol for the Cavernomas: A Randomised Effectiveness (CARE) pilot trial
Source: BMJ Open. 2023 Aug 9;13(8):e075187. doi: 10.1136/bmjopen-2023-075187 (PMC10414059; doi:10.1136/bmjopen-2023-075187)
Supplement: Supplementary data [file bmjopen-2023-075187supp003.zip › 02 PIL & CF/CARE - Supplementary PIL (Adult) V2.0 22Mar2021 Clean.docx]

**C**avernomas: **A** **R**andomised **E**ffectiveness (**CARE**) Trial

**Supplementary Information for Adults**

Contents of this leaflet:

Why do we need to undertake this research? 2

What is this research study about? 5

Why have I been invited to take part? 5

Do I have to take part? 6

What will happen if I take part? 7

What are the possible benefits of taking part? 12

What are the possible disadvantages and risks of taking part? 13

Is there anything I need to do or avoid? 14

What if there are any problems? 14

What if new information becomes available? 14

What will happen if I don’t want to carry on with the study? 15

Will my taking part be kept confidential? 15

How will we use information about you? 16

What about future research? 17

How long will you store my audio-recording and interview data for? 18

Where can you find out more about how your information is used? 18

What will my DNA sample be used for? 19

What happens when the study is finished? 19

What will happen to the results of the study? 20

Who is organising and funding the research? 20

Who has reviewed the study? 21

Contact details 22

**Thank you for reading the Short Information Leaflet for Adults**

**This leaflet provides more information about the research**

In the Short Information Leaflet, we invited you to take part in this research study. We explained why you have been invited. We provided you with some background about brain cavernoma and its treatment. We explained why we need to undertake this research, and outlined what will happen if you take part. This leaflet provides more information about what the research involves, to help you to decide whether to take part.

## Why do we need to undertake this research?

The risks and benefits of **treatment without surgery** seem similar to those of **treatment including surgery** for people with brain cavernoma. There is no reliable evidence about which is better. This can leave doctors and patients uncertain about whether to use surgery. Answering this uncertainty is the top priority for research into cavernoma.

**Treatment without surgery** of a brain cavernoma that has caused symptoms may involve treating your symptoms, preventing seizures, rehabilitation and brain scans.

**Treatment including surgery** of a brain cavernoma that has caused symptoms also involves trying to remove the cavernoma using brain surgery (known as **neurosurgery**) or trying to stabilise the cavernoma using focussed radiation treatment (known as **stereotactic radiosurgery**). In this study, the type of stereotactic radiosurgery will be Gamma Knife, given in Sheffield or London.

Neurosurgery and stereotactic radiosurgery are quite different. What’s involved, and the possible benefits and risks of the two procedures, are described in the table on the next two pages. A full description of these procedures should have been provided by the doctor looking after you. More information is available from Cavernoma Alliance UK ([www.cavernoma.org.uk](http://www.cavernoma.org.uk)).

|  | **Treatment without surgery** | **Treatment including surgery** | |
| --- | --- | --- | --- |
|  |  | **Neurosurgery** | **Stereotactic radiosurgery** |
| **What may be involved?** | - Treat symptoms - Prevent seizures - Rehabilitation - Brain scan | - Treat symptoms - Prevent seizures - Rehabilitation - Brain scan | - Treat symptoms - Prevent seizures - Rehabilitation - Brain scan |
|  |  | - Hospital admission for days - General anaesthetic - Opening in the skull - Operation to remove cavernoma - Follow-up brain scan - Must not drive for 6 months | - Hospital attendance for a day - Anaesthetic not needed - Head fixed in a temporary frame - Focussed radiation given once - Follow-up brain scans |
| **What are the possible benefits?** | - Bleed/stroke risk reduces as time passes - Avoids risks of neurosurgery or radiosurgery | - Risk of bleed/stroke lower if cavernoma removed - Less worry about symptoms returning | - Risk of bleed/stroke may be lower if cavernoma stabilised, but these benefits are uncertain - Less worry about symptoms returning |
| **What are the possible risks?** | - Future bleed/stroke due to cavernoma - Can be mild - May be disabling - Rarely be fatal - Risk higher for cavernoma in brainstem | - Bleed/stroke due to neurosurgery - Can be mild - May be disabling - Rarely be fatal - Risk higher for cavernoma in brainstem | - Bleed/stroke despite radiosurgery - Can be mild - May be disabling - Rarely be fatal - Risk higher for cavernoma in brainstem |
|  | - Epileptic seizures, which may be difficult to control | - Epileptic seizures may not go away | - Epileptic seizures may not go away |
|  | - Cavernoma remains in the brain, so the risks of stroke and seizure may never go away | - Cavernoma may come back | - Cavernoma not removed |
|  | - Worry about symptoms returning | - Complications of treatment (e.g. infection or damage to brain around the cavernoma) | - Complications of treatment (e.g. damage to brain around the cavernoma) |

## What is this research study about?

You can take part in either or both of the two parts of this study:

- **The Randomised Study** is comparing treatment without surgery to treatment including surgery for people with symptoms from brain cavernoma.

We will put everyone who takes part in the Randomised Study into one of two groups in a randomised controlled trial. One group will receive treatment without surgery. The other group will receive treatment including surgery. The only thing that should differ between the two groups is the type of treatment.

To make sure the groups are as similar as possible to start with, people will be put into a group randomly (by chance).  The chance of being in either group is 1 in 2 (or 50% or 50:50).

You and your doctor or surgeon will not be able to choose which of these groups you go into. This is to ensure that the results of the study are not influenced (biased), for example if younger, fitter people are more likely to choose one group over the other.

This Randomised Study is a ‘pilot study’ that will tell us if enough eligible patients take part to indicate that a larger randomised trial could be done to determine whether treatment without surgery or treatment including surgery is best.

- **The Information Study** looks at how information is given to patients and interviews them to find out what they think about taking part.

You can take part in either or both parts of this study.

## Why have I been invited to take part?

People of any age or sex are asked to take part because:

- You have had symptoms caused by a brain cavernoma.
- Your doctor is not sure whether to recommend that you should have treatment without surgery, or treatment including surgery.
- You are able to make decisions for yourself.
- You have at least one brain cavernoma that has not been treated with surgery.

## Do I have to take part?

No, it’s up to you to decide whether to take part in either of the two parts of the study. Your decision will not affect your standard medical care. If you do not want to take part in the Randomised Study, we are still interested to interview you in the Information Study to understand why. Your feedback will help us design the CARE trial and future studies. Let the research team know if you are happy to be interviewed. The diagram below summarises your options.


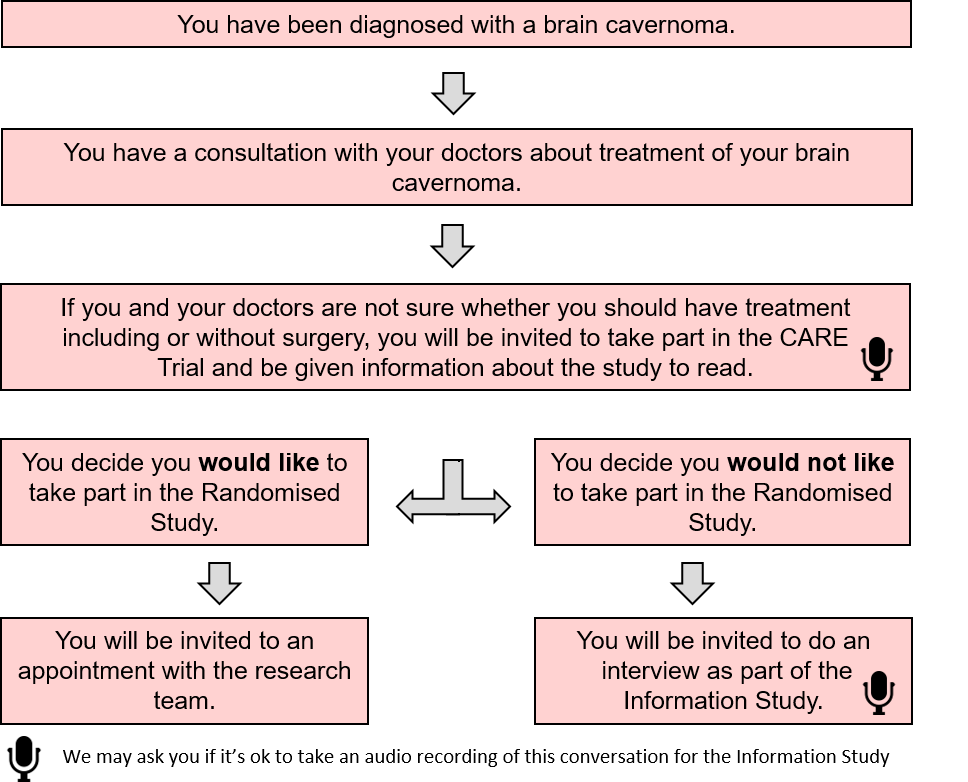


## What will happen if I take part?

If you decide to take part you will be given this information leaflet to keep and you will be asked to sign a consent form. You are free to withdraw at any time and without giving a reason. The research doctor or nurse will go through this information leaflet with you and answer any questions you have. You can take as long as you need to ask questions and decide. If you are happy to take part, you will be asked to sign a consent form on which you can mark which parts of the research you would like to take part in.

### The Randomised Study

If you are interested in taking part in the Randomised Study:

- We will arrange a **research appointment** with you. You can, but do not have to come to the hospital for this. If you prefer not to come to the hospital, a member of your local research team (a doctor or nurse involved in the study) will get in touch with you by telephone or another remote video call contact method (e.g. NHSNearMe). The appointment should last around 1 hour. At this appointment:
  - You will be asked to sign a consent form, and a researcher will complete some **questionnaires** about you to **provide information about your cavernoma diagnosis, your medical history and the medicines** you are taking.
  - If you come to the hospital for the appointment, you will be asked to provide a small blood sample. This is optional and will be used in the future to look at your DNA.
- You will then be **allocated at** **random** by a computer to either treatment without surgery or treatment including surgery. You will be told straight away which one you will receive.
  - You and your hospital doctor will have already agreed whether neurosurgery or stereotactic radiosurgery would be most suitable if you’re allocated to treatment with surgery.
  - If you and your doctor weren’t sure about the best type of surgery for you, this will also be allocated at random.
  - If you’re allocated to **treatment including surgery**, you will be added to the waiting list for either neurosurgery or stereotactic radiosurgery, according to what was agreed with your doctor.
  - If you are allocated to **treatment without surgery**, your care will continue unchanged.
- The doctor or nurse will take an electronic copy of the **brain scans** that diagnosed your brain cavernoma from your medical records. These will be kept with the research data for the study.
- The doctor or nurse will arrange for you to **come to the hospital** **6 months after your first appointment** to see a member of your local research team to see how you are doing. You will be asked to have a **brain MRI scan** around the time of this appointment and fill in some more questionnaires. The brain MRI scan will be used to assess the cavernoma and whether it has changed since you were diagnosed. This scan will be extra to your normal clinical care if you are treated without surgery. The scan will take 20-30 minutes. You will be invited to provide a small blood sample if you haven’t already. The whole appointment should last around 3 hours.
- We will reimburse reasonable travel expenses incurred by attending the hospital for research appointments.
- A member of the central research team (based within the Trial Coordinating Centre at the Edinburgh Clinical Trials Unit) will get in touch with you by **telephone or email every 6 months** until the end of the study to check how you are doing. They will send you some questionnaires by post for you to post back to them by freepost. We currently plan to finish the study around May 2023 but it may be later if further funding is obtained to continue, in which case we’ll let you know.
- We will also take an electronic copy of any other relevant **brain scans** that you have during the study for example any scans done in relation to surgery or if you experience any problems such as a bleed. These will be kept with the research data for the study.
- We will ask for your permission to continue collecting information about your health from your medical records after you are finished in the study. You can tell us if you do not want us to do this.

What’s involved in the Randomised Study is illustrated in the diagram on the next page.


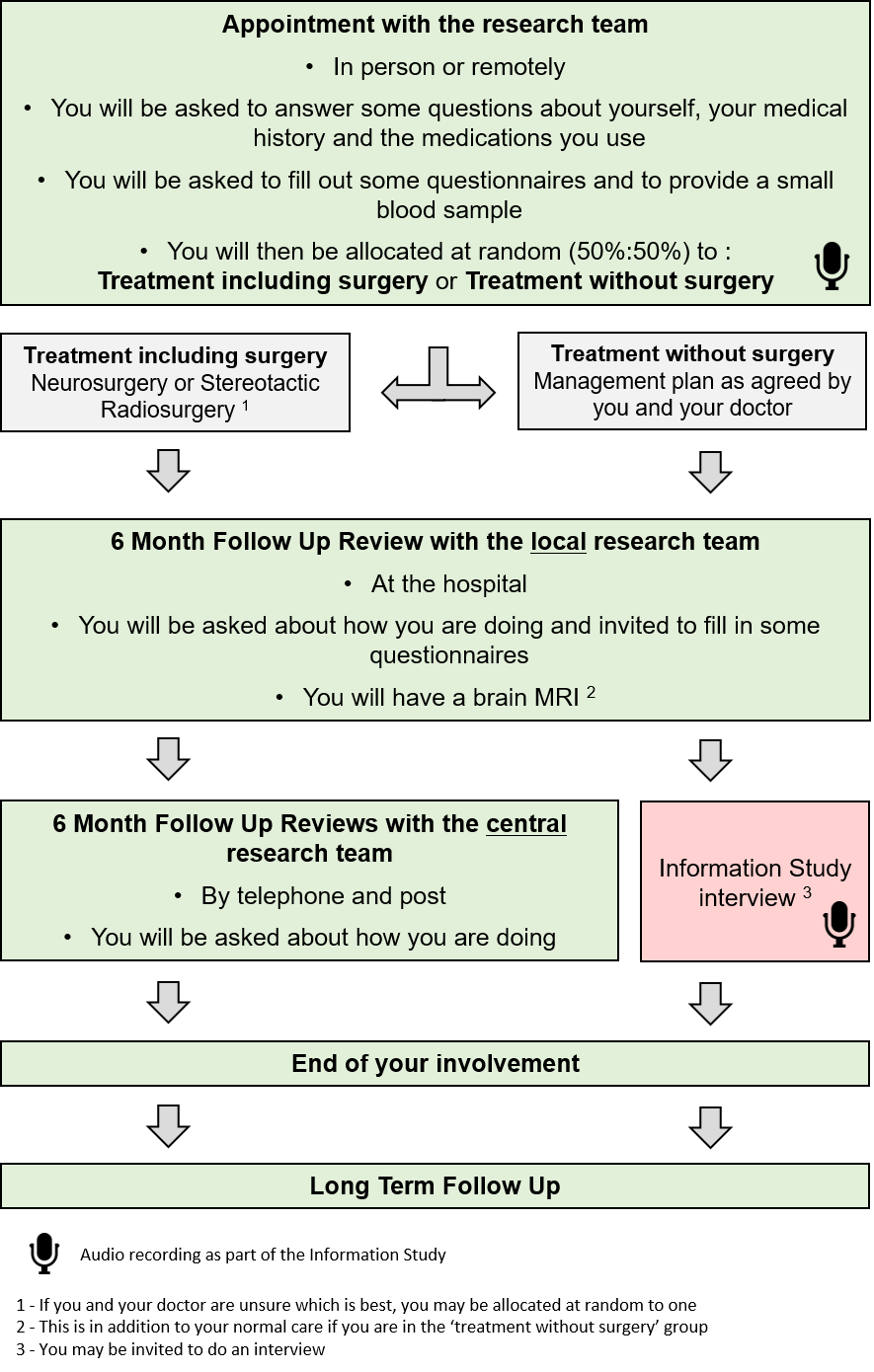


These links provide useful information about randomised trials:

| 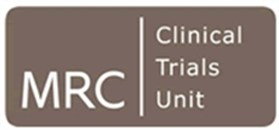 | Five-minute video, “The Gold Standard. What are randomised controlled trials and why are they important?” |
| --- | --- |
| <https://youtu.be/U6kVlRn6G0w> | |
| 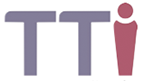 | Read a patient-friendly guide to key concepts for critical thinking about treatment claims. Or listen to the audio book. |
| [www.testingtreatments.org](http://www.testingtreatments.org) | |
| 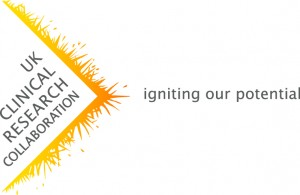 | Read a short pamphlet, “Clinical trials: what they are and what they’re not.” |
| [www.ukcrc.org/wp-content/uploads/2014/03/iCT_leaflet.pdf](http://www.ukcrc.org/wp-content/uploads/2014/03/iCT_leaflet.pdf) | |
| 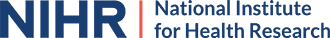  Be Part of Research | NIHR Be Part of Research can help you make informed choices about clinical trials. |
| <https://bepartofresearch.nihr.ac.uk/> | |
| 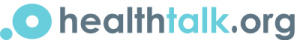 | 42 videos of people talking about their experiences of taking part in a clinical trial. |
| <https://healthtalk.org/clinical-trials/overview> | |

### The Information Study

- This part of the research will look at what information people need to decide whether to take part in the CARE trial.
- To find this out we may ask if we can audio-record the video, telephone or face-to-face appointments that take place between patients and healthcare staff, during which this study is discussed. This will allow us to understand and improve how research is discussed with patients.
- We will also invite some patients to take part in an interview with one of the University of Bristol’s researchers to ask about their experience of being invited to take part in the CARE trial. These interviews will be done using Zoom or a similar programme, or by telephone. The interview will last approximately 45 minutes. The researcher will contact you to find a mutually convenient time to do the interview.

You can agree to the Randomised Study, the Information Study, or both. You can decline to take part in the Information Study whilst still being able to take part in the Randomised Study and vice versa.

If you decide that you do not want to take part in any aspect of the study, we would be interested in interviewing you to understand why. This will help us to improve the design of future studies.

## What are the possible benefits of taking part?

There are various benefits to taking part in this research study:

- You may find it a relief to have the decision about whether to have surgery taken out of your hands.
- Your health in this study will be under review with the possibility of an additional brain MRI scan. You may feel supported by this.
- The results of this study will help us to improve the healthcare of patients in the future.

Your participation in the Randomised Study will help us to work out if it will be possible to determine whether treatment without surgery or treatment including surgery is best in a larger study in the future. Your participation will allow us to design future studies with patients, carers and families at the forefront.

Your participation in the Information Study will allow us to improve how cavernoma treatment research is discussed with patients.

## What are the possible disadvantages and risks of taking part?

The risks associated with treatment without surgery and treatment including surgery are summarised on pages 3 and 4 of this leaflet.

If you take part in this study you may have stereotactic radiosurgery. This procedure uses ionising radiation to provide treatment. Ionising radiation can cause cell damage that may, after many years or decades, turn cancerous. Taking part in this study will not significantly alter the chances of this happening to you. We are all at risk of developing cancer during our lifetime. The normal risk is that this will happen to about 50% of people at some point in their life. Taking part in this study will increase the chances of this happening to you from 50% to between 50 and 50.5%.

If you have a brain MRI scan, you might become claustrophobic in the MRI scanner – but if that happened to you, the scan would be stopped. Having another brain MRI scan runs a very low risk of detecting other problems that you have no symptoms of. Whilst this may sound like a good thing it can lead to unnecessary anxiety or more tests. We will not inform you about any minor findings that will not cause you any problems, but if we see a problem on the scan that might affect your health, then we will notify your cavernoma consultant who will arrange appropriate follow up and we will also let your GP know about this. If you have had a scan recently, it is unlikely that we will find anything new unexpectedly.

Attending research appointments at the hospital and completing the 6-month follow up questionnaires at home will take up some of your time. The researchers involved in the study will do their best to work around you when organising appointments and calls.

Taking a blood sample for DNA may cause discomfort or bruising.

Taking part in an interview may involve the discussion of sensitive issues; however, the research team and interviewer will be fully trained and will support you during these discussions. Completing the interview will also take up some of your time.

## Is there anything I need to do or avoid?

The brain MRI scan you will receive is noisy but does not involve any radiation. MRI is safe as long as standard precautions are taken to remove metal from your person. The radiographer who operates the scanner will check you for metal before you go into the scanner. Some people with certain types of medical implants (pacemakers, cochlear implants, aneurysm clips inside the head) cannot have an MRI scan because the magnet might damage the implant.

If you take part in any other research studies, we ask that you let your local research team know.

## What if there are any problems?

If you have a concern about any aspect of this study please contact [INSERT RESEARCH TEAM STAFF NAME AND CONTACT DETAILS] who will do their best to answer your questions.

In the unlikely event that something goes wrong and you are harmed during the research and this is due to someone’s negligence, then you may have grounds for a legal action for compensation against NHS [INSERT BOARD/TRUST NAME] but you may have to pay your legal costs. The normal National Health Service complaints mechanisms will still be available to you (if appropriate).

## What if new information becomes available?

If new information becomes available which might influence whether you should continue to take part in the study, the research team will contact you.

## What will happen if I don’t want to carry on with the study?

Your decision to take part in the study is entirely voluntary. You (or your legal representative if you were to lose mental capacity during the study) are free to withdraw from all or part of this study at any time, without giving a reason. Any decision to withdraw will not affect the standard medical care that you receive.

If you decide to withdraw, we will retain information collected about you before you withdrew. We need to manage your records in specific ways for the research to be reliable. This means that we may not be able to let you see or change the data we hold about you. If we have not yet analysed your DNA sample, you can request that we destroy this.

If you decide to withdraw from the Randomised Study, you can still take part in the Information Study and an interview with the researcher to discuss your experiences.

If you choose to stop taking part in the Randomised Study, we would like to continue collecting information about your health from your medical records. You can tell us if you do not want this to happen. We would also like your permission to continue to collect information relevant to your cavernoma treatment from your medical records after the study has finished on a long-term basis. We will use your NHS number (or equivalent in other nations) to do this. If you do not want this you can tell us and we will not do so.

If you withdraw, please contact your local research doctor or nurse to indicate which parts of the study you are withdrawing from. You can find all of the contact details you need on the last page of this leaflet.

## Will my taking part be kept confidential?

All the information we collect during the course of the research will be kept confidential. There are strict laws which safeguard your privacy at every stage. We will keep all information about you safe and secure. People will use your information to do the research or to check your records to make sure that the research is being done properly.

## How will we use information about you?

### Randomised Study

We will need to access your medical records to conduct this study. We will use information collected from you and from your medical records. This information includes your name, date of birth, address and NHS number (or equivalent in other nations). Only authorised personnel will access your medical notes as part of the study. People who do not need to know who you are will not be able to see your name or contact details. Your data will be labelled with a code number (unique study identifier) instead.

Your contact details will be used by members of your local research team and the central research team at the University of Edinburgh to organise appointments with you and to do follow-up reviews.

With your permission, we would like to inform your GP of your participation in this study. We will also notify you and your GP should anything clinically important come to light during the study.

Your information will be held at the hospital by the research team. Information that is collected on paper forms will be stored securely (i.e. in a locked cabinet with restricted access). Your information, including your NHS number (or equivalent in other nations) and contact details, will also be entered into an electronic database developed and maintained by the Trial Coordinating Centre at the Edinburgh Clinical Trials Unit, University of Edinburgh. The database is held on a server at the University of Edinburgh, has restricted access and is password protected so that only approved personnel can access it. The team managing the study at the University of Edinburgh will have access to the information collected for administrative purposes.

In order to monitor and audit the study we will ask your consent for representatives from the Sponsor and NHS Institutions to access your medical records and data collected during the study, where it is relevant to you taking part in this research. The Sponsor is the body that is responsible for overall management of the study and providing insurance and indemnity.

### Information Study

The audio-recordings of your conversations with the research team about recruitment to the study will be securely passed to the researchers at the University of Bristol. We will not put your name or any other personal information that might identify you on any recordings. The data will be labelled with a code number (unique study identifier) instead.

If you have consented to do an interview, the qualitative researcher at the University of Bristol will use your contact details to get in touch with you to do the interview which will be recorded. The audio-recording will be stored on a secure server at the University of Bristol. Only the qualitative researcher or other approved personnel working on the study will have access to this storage area.

All recordings will be listened to by the qualitative researcher or other approved personnel and transcripts (written versions) of the conversations prepared by either an approved University of Bristol employee or a third-party transcribing service approved by University of Bristol. These transcripts will not have your name or other personal details on. Any paper copies of transcripts or other study documentation will be stored securely (i.e. in a locked cabinet with restricted access).

## What about future research?

If we share any of the data collected as part of the study, we will only share what’s called “de-identified” data and will only do so with your consent. This means that any data that are shared would not include any personal details and could not be used to identify you. Sharing of your data would be overseen by the University of Edinburgh, in line with their strict rules of confidentiality. The Chief Investigator of the study, Professor Rustam Al-Shahi Salman will be responsible for ensuring that your data are held securely.

Your study data (from the Randomised Study and Information Study), brain scans, and DNA sample will be used in future studies and may be shared with researchers from other institutions. We may use the qualitative data collected (including quotes) in future research, teaching and publications looking at common issues across studies. You will not be identified in any way in any presentation, report or publication. The data generated from your participation in this study may also be shared in the future with groups such as the funder, study collaborators and/or as part of the publication process.

## How long will you store my audio-recording and interview data for?

At the end of the study, audio-recordings will be kept for at least 10 years before they will be destroyed. Transcripts will be stored indefinitely in secure research data storage, which can be accessed by approved individuals who are interested in conducting their own analyses of the data. These individuals will have to submit an application to do this, which will be assessed by an independent committee. However, all data will have identifiable information removed before they are made available, and there will be no way to identify you or any other individuals mentioned in your interviews/appointments. Sharing access to research data and findings is considered good research practice and is a requirement of many funding bodies and scientific journals. Sharing data helps to maximise the impact of money invested into conducting research studies and can encourage new avenues of research.

## Where can you find out more about how your information is used?

You can find out more about how we use your information:

- at [www.hra.nhs.uk/information-about-patients/](https://www.hra.nhs.uk/information-about-patients/)
- in a leaflet available at [www.hra.nhs.uk/patientdataandresearch](http://www.hra.nhs.uk/patientdataandresearch)
- by asking one of the research team (contact details are at the end of the leaflet)
- by sending an email to [dpo@ed.ac.uk](mailto:dpo@ed.ac.uk) (University of Edinburgh Data Protection Officer)
- by ringing us on [RESEARCH TEAM PHONE NUMBER].

## What will my DNA sample be used for?

We will use the DNA sample (which contains your genetic code) you provide to investigate whether it affects the outcome of treatment including surgery. We may also contribute DNA to other research related to brain cavernoma in the future. Separate approvals will be sought by the researchers doing other research in the future. You can indicate on the consent form if you would like to be informed about the results of research using your DNA sample that are relevant to your health.

## What happens when the study is finished?

Whether you are allocated to treatment without surgery or treatment including surgery, the care that you receive during and after the study will be your normal medical care and this will continue as usual after the study ends.

After the study has finished, we would like to be able to access your health records so we can continue to enquire about your long-term health status and collect additional information relevant to the study. We will ask you to consent to this. We’ll periodically look at your electronic health records and ask central NHS registers to give us information about your health. To do this, we’ll need to collect your unique patient identifier (NHS number or equivalent in other nations) and store it at the University of Edinburgh.

We plan to keep records of your personal details, the blood sample you provided and the data that we have gathered about you during the study for an indefinite period of time. The reason for this is that they may be of great value in advancing understanding of the causes and treatment of cavernoma in future research.

## What will happen to the results of the study?

We hope to have the result of the study within the next 5 years. The results of the study will help us to decide whether we can conduct a large-scale randomised study comparing treatment without surgery to treatment including surgery for patients with symptomatic brain cavernoma. The results will be published in scientific journals and presented at conferences.

We will not use your name in any reports or presentations of the study findings or reveal that you were interviewed. We may use quotes and play parts of your audio-recordings (from interviews and appointments/meetings) as part of publications, teaching and presentations at academic meetings. If we do use any of your data, all quotes will be de-identified (and voices modified if necessary) so that you cannot be identified.

Once the study has been published, a summary of the findings will be available on the Edinburgh Clinical Trials Unit website ([www.ed.ac.uk/edinburgh-clinical-trials](http://www.ed.ac.uk/edinburgh-clinical-trials)). You can also request a summary of the results through a member of the research team. We may also contact you to invite you to a presentation of the results and to give your feedback on the study.

## Who is organising and funding the research?

This study is funded by the National Institute for Health Research (NIHR) and is led by Professor Rustam Al-Shahi Salman, Professor of Clinical Neurology at the University of Edinburgh. Some of the research team’s salaries are funded by the NIHR award for this study. Cavernoma Alliance UK and the University of Bristol are key collaborators involved in the study. The study is sponsored by the University of Edinburgh and NHS Lothian.

## Who has reviewed the study?

The study proposal has been reviewed by neurologists and neurosurgeons from institutions in Edinburgh, London, Sheffield, Newcastle, Liverpool and Cambridge. It has also been reviewed by the qualitative research group at the University of Bristol. The study proposal was reviewed extensively by experts at the NIHR.

The charity Cavernoma Alliance UK (CAUK) has been involved in all steps of the development of the study proposal. The question at the core of this study was identified through a member-supported Priority Setting Partnership (James Lind Alliance) with the charity. As part of developing the proposal, the Chief Investigator worked with CAUK to gather insights from patients, carers and families. These insights have shaped the design of the study. CAUK have set up a patient advisory group specifically for the CARE trial and they provide input on all aspects of set-up and running of the study. The group will also review and provide feedback on the results of the study when they are available.

All research in the NHS is looked at by an independent group of people, called a Research Ethics Committee. The Yorkshire & The Humber - Leeds East Research Ethics Committee, which has responsibility for scrutinising proposals for medical research on humans, has examined the proposal and has raised no objections from the point of view of research ethics. NHS Management Approval has also been given.

## Contact details

### Researcher Contact Details

If you have any further questions about the study please contact research team member [NAME] on [PHONE NUMBER] or email on: [EMAIL ADDRESS].

### Cavernoma Alliance UK Contact Details

If you would like to discuss this study with a member of the Cavernoma Alliance UK Patient Advisory Group, you can contact them using the details below:

*TO BE ADDED BEFORE USE AT SITES:*

[NAME]

[EMAIL ADDRESS]

[PHONE NUMBER]

### Independent Contact Details

If you would like to discuss this study with someone independent of the study please contact:

Dr Jonathan Berg

Senior Lecturer and Honorary Consultant in Clinical Genetics

NHS Research Scotland Lead for Genetics and Rare Diseases

Ninewells Hospital and Medical School

University of Dundee

Tel: 0044 (0) 1382 425716 / Email: [j.n.berg@dundee.ac.uk](mailto:j.n.berg@dundee.ac.uk)

### Complaints

If you wish to make a complaint about the study please contact:

[INSERT CONTACT DETAILS] *to be adapted depending on research site*

**Thank you for reading this supplementary information leaflet and considering participating in this research study**
