# Supplementary material for: Feasibility of comparing medical management and surgery (with neurosurgery or stereotactic radiosurgery) with medical management alone in people with symptomatic brain cavernoma – protocol for the Cavernomas: A Randomised Effectiveness (CARE) pilot trial
Source: BMJ Open. 2023 Aug 9;13(8):e075187. doi: 10.1136/bmjopen-2023-075187 (PMC10414059; doi:10.1136/bmjopen-2023-075187)
Supplement: Supplementary data [file bmjopen-2023-075187supp003.zip › 02 PIL & CF/CARE - Letter for Adult Participant Representative (LoC) V1.0 (27Jan2021).docx]

**C**avernomas: **A** **R**andomised **E**ffectiveness (**CARE**) Trial

**Information for Representatives of Adult Participants who have lost mental capacity**

The participant you are representing previously provided their consent to take part in this research study. We would like to confirm with you as their representative* that they should continue to take part while they don’t have the mental capacity to make decisions for themselves.

Under these circumstances, we are inviting you as a representative of the participant to complete a Consultation Form on their behalf. This form has two parts, and you are asked to complete EITHER Part A OR Part B.

Part A is for when you do not wish to amend the consent form previously provided by the participant, and Part B is when you do wish to amend that consent form.

To help you decide, please take time to read the attached information leaflet about why the research is being done and what it will involve. Talk to others about the study if you wish and ask if there is anything that is not clear or if you would like more information.

We would ask that you put aside your own views about the research and consider the previous decision made by the participant, as well as what you think their present feelings and wishes would be, if they were able to consent for themselves.

If you decide that the participant you represent would not wish to continue to take part, it will not affect the standard of care they receive in any way.

If/when the participant who you are representing has regained mental capacity and is able to consent for themselves again, we will confirm with them that they are happy to continue to take part.

If you have any questions please feel free to contact a member of the research team (details at the end of the Patient Information Leaflet).

***Thank you for your time***

*Definition of ‘representative’:*

*In Scotland, patient representatives may be a nearest relative, welfare guardian or welfare attorney who is authorised to take decisions about the research.*

*In the rest of the UK, a consultee should be sought (defined as an unpaid person with an interest in the welfare of the participant, who is willing to help). If no appropriate person can be identified who is willing to act as a personal consultee, the researcher may consult a “nominated consultee”, i.e. a person independent of the project appointed in accordance with the Department of Health’s guidance on nominating a consultee for research involving adults who lack capacity to consent.*

*In the Republic of Ireland, a person not connected with the conduct of the trial who by virtue of his/her family relationship with an adult lacking decision-making capacity, is suitable to act as the legal representative and is willing and able to do so or (if there is no such individual) a person who is not connected with the conduct of the trial, who is a solicitor nominated by the relevant health care provider.*
