# Supplementary material for: Feasibility of comparing medical management and surgery (with neurosurgery or stereotactic radiosurgery) with medical management alone in people with symptomatic brain cavernoma – protocol for the Cavernomas: A Randomised Effectiveness (CARE) pilot trial
Source: BMJ Open. 2023 Aug 9;13(8):e075187. doi: 10.1136/bmjopen-2023-075187 (PMC10414059; doi:10.1136/bmjopen-2023-075187)
Supplement: Supplementary data [file bmjopen-2023-075187supp003.zip › 02 PIL & CF/CARE - Consent Form (Parent Guardian) V2.0 22Mar2021 Clean.docx]

**CARE pilot trial (Randomised Study)**

**Parent/Guardian Informed Consent Form**

| Please **initial** box | |
| --- | --- |
| 1. I confirm that I have read and understand the information sheet (Version Number:___, Date:____________) for the CARE pilot trial. I have had the opportunity to consider the information, ask questions and have had these questions answered satisfactorily. |  |
| 1. I understand that my child/ward’s participation is voluntary and that I am free to withdraw them at any time, without giving any reason and without my child/ward’s medical care and/or legal rights being affected. |  |
| 1. I give permission for the research team to access my child/ward’s medical records for the purposes of this research study. |  |
| 1. I understand that relevant sections of my child/ward’s medical notes and data collected during the study may be looked at by individuals from the Sponsor (University of Edinburgh and/or NHS Lothian), from regulatory authorities or from the NHS organisation where it is relevant to them taking part in this research. I give permission for these individuals to have access to my child/ward’s data and/or medical records. |  |
| 1. I give permission for my personal information and my child/ward’s (including name, address, email address, date of birth and telephone number) to be entered into an electronic database managed by the Edinburgh Clinical Trials Unit and that relevant staff will have access to this information for administration purposes. I understand that my contact details will be used to contact me about my child/ward’s participation in the study. |  |
| 1. I give permission for my child/ward’s NHS or Community Health Index (CHI) number or equivalent in other nations, and hospital number to be collected and passed to the Edinburgh Clinical Trials Unit. |  |
| 1. I agree to my child/ward’s General Practitioner being informed of their participation in the study and providing follow-up information about their current and future health problems for the duration of the study follow up period. |  |
| 1. I understand that data collected about my child/ward during the study may be converted to de-identified data. |  |
| 1. I agree that the researchers can find out how my child/ward is getting on by contacting me every 6 months for the duration of the study follow up period. |  |
| 1. My child/ward’s relatives, carers, or close personal contacts named on the Contact Form are willing to provide information about how they are getting on. These people or others caring for them may provide this information if I cannot be contacted or I am no longer able to make decisions for myself. |  |
| 1. I agree to my child/ward giving a blood sample which will be used for genetic (DNA) analysis. | Yes No |
| 1. If my child/ward’s DNA sample is analysed in the future, I would like to be informed about any results that are relevant to their health. | Yes No |
| 1. I agree to the following being shared with other researchers and being used in future studies, whatever happens to my child/ward:  - De-identified data - De-identified brain imaging - De-identified blood or DNA sample | Yes No  Yes No  Yes No |
| 1. I understand that the information held and maintained by NHS Digital and other central UK NHS bodies (or the equivalent in other nations) may be used to help contact me or provide information about my child/ward’s health and use of services. I agree to my child/ward’s NHS or Community Health Index (CHI) number or the equivalent in other nations being used to gather information relevant to their participation after the study has finished. | Yes No |
| 1. I would like to be informed of the results of the CARE pilot trial. | Yes No |
| 1. I agree to my contact details being kept on record and am happy to be contacted about similar research in the future. | Yes No |
| 1. I agree to my child/ward taking part in the above study. |  |

|  |  |  |  |  |
| --- | --- | --- | --- | --- |
| Name of Person Giving Consent |  | Date |  | Signature |
|  |  |  |  |  |
| Witness (if participant has provided verbal consent) |  | Date |  | Signature |
|  |  |  |  |  |
| Name of Person Receiving Consent (researcher) |  | Date |  | Signature |

1x original – into Site File; 1x copy – to Participant; 1x copy – into medical records
