# Supplementary material for: Feasibility of comparing medical management and surgery (with neurosurgery or stereotactic radiosurgery) with medical management alone in people with symptomatic brain cavernoma – protocol for the Cavernomas: A Randomised Effectiveness (CARE) pilot trial
Source: BMJ Open. 2023 Aug 9;13(8):e075187. doi: 10.1136/bmjopen-2023-075187 (PMC10414059; doi:10.1136/bmjopen-2023-075187)
Supplement: Supplementary data [file bmjopen-2023-075187supp003.zip › 02 PIL & CF/CARE - Information Study Verbal Consent to Audio Rec Form V1.0 09Dec2020.docx]

***To be completed by the recruiter NOT FOR PATIENT COMPLETION***

Verbal Consent to Audio-Recording

CARE Information Study

| **Study Title:** | Cavernomas A Randomised Effectiveness (CARE) pilot trial, to address the effectiveness of active treatment (with neurosurgery or stereotactic radiosurgery) versus conservative management in people with symptomatic brain cavernoma |
| --- | --- |
| **Study Identification Number:** | IRAS 289197 / [REC REF] |
| **Patient Initials:** |  |
| **Patient Screening / Identification Number:** |  |

I confirm the patient or parent/guardian verbally consented to having their appointment audio-recorded for training and research purposes

**Please initial box:**

Please provide the patient with the current version of the **CARE Trial Patient Information Leaflet** (if not already received).

**Should the patient decide not to sign the “Audio-recording of consultations” Consent Form all recordings must be destroyed. No recordings may be transmitted to the University of Bristol unless the patient has signed the consent form.**

| **Investigator Statement and Signature**  ***To be completed by the investigator or designee taking verbal consent*** | | |
| --- | --- | --- |
| Signed:  _________________________________ | Date:  ________________ | Name in block letters:  _____________________________________ |

1 copy for local site file; 1 to be kept with hospital notes
