# Supplementary material for: Feasibility of comparing medical management and surgery (with neurosurgery or stereotactic radiosurgery) with medical management alone in people with symptomatic brain cavernoma – protocol for the Cavernomas: A Randomised Effectiveness (CARE) pilot trial
Source: BMJ Open. 2023 Aug 9;13(8):e075187. doi: 10.1136/bmjopen-2023-075187 (PMC10414059; doi:10.1136/bmjopen-2023-075187)
Supplement: Supplementary data [file bmjopen-2023-075187supp003.zip › 02 PIL & CF/CARE - Childrens 0-5 years PIL V1.0 07Dec2020.docx]

**Information Leaflet for children 5 years old and younger**

| **Would you and your mummy or daddy or the grown up who looks after you be able to help us?** |
| --- |
| The doctor has said that there is a problem with your brain that is making you poorly. The doctor might give you medicine. Sometimes doctors also do an operation to fix the problem with your brain. But sometimes they don’t.  We want to find out which is best: taking medicine and doing an operation or taking medicine.  **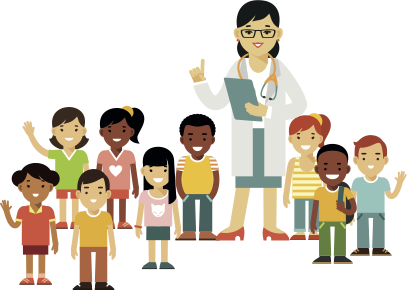**  We are asking lots of other children and grown-ups to help us too. Your doctor will give different things to different people and see which works best. |
| If you want to help us |
| You can help if you want to. Your mummy or daddy or the grown up who looks after you will need to say that it is okay. |
| **This might help us help other children who have problems with their brain too.** |

*Illustrations by Getty Images*
