# Supplementary material for: Feasibility of comparing medical management and surgery (with neurosurgery or stereotactic radiosurgery) with medical management alone in people with symptomatic brain cavernoma – protocol for the Cavernomas: A Randomised Effectiveness (CARE) pilot trial
Source: BMJ Open. 2023 Aug 9;13(8):e075187. doi: 10.1136/bmjopen-2023-075187 (PMC10414059; doi:10.1136/bmjopen-2023-075187)
Supplement: Supplementary data [file bmjopen-2023-075187supp003.zip › 02 PIL & CF/CARE - Young Persons PIL AF (11-15yrs) V1.0 08Dec2020.docx]

**Young Person’s Information Leaflet and Assent Form (11-15 years old)**

| We are inviting you to take part in a research study.  To help you decide whether or not to participate, it is important that you understand why the study is being done and what it will involve. Please read and think about this leaflet carefully. Talk to your family, friends, doctor or nurse about it if you want.  If something isn’t clear or if you have more questions, you can ask your parents or guardian to give a doctor or nurse involved in the study at your hospital a call and we can discuss it with you and your parent or guardian.  Thank you for reading this**.**  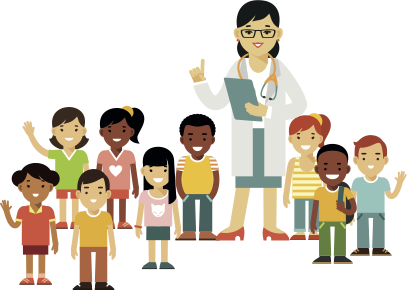 |
| --- |
|  |
| What is the purpose of the study? |
| We are trying to find out the best type of treatment for people with brain cavernoma. A cavernoma is made up of abnormal blood vessels and can be found in the brain and/or spinal cord and looks like a raspberry. There are two kinds of treatment to choose from:   1. **Treatment without surgery.** This may involve taking medicines or other treatments such as exercises and activities that will help you feel better if you’ve been poorly 2. **Treatment including surgery.** This involves surgery to remove the cavernoma (neurosurgery) or a kind of surgery using radiation (radiosurgery) to stabilise the cavernoma and may also involve taking medicines or other treatments.   There are risks and benefits associated with both kinds of treatment and we don’t know which is best. There has been no proper research to answer this question.  The best way to find out is to do a **research study**. |
| Why have I been chosen? |
| We asked you because you have a brain cavernoma and are going to receive treatment but your doctor is not sure which treatment is best for you. |
| Do I have to take part? |
| 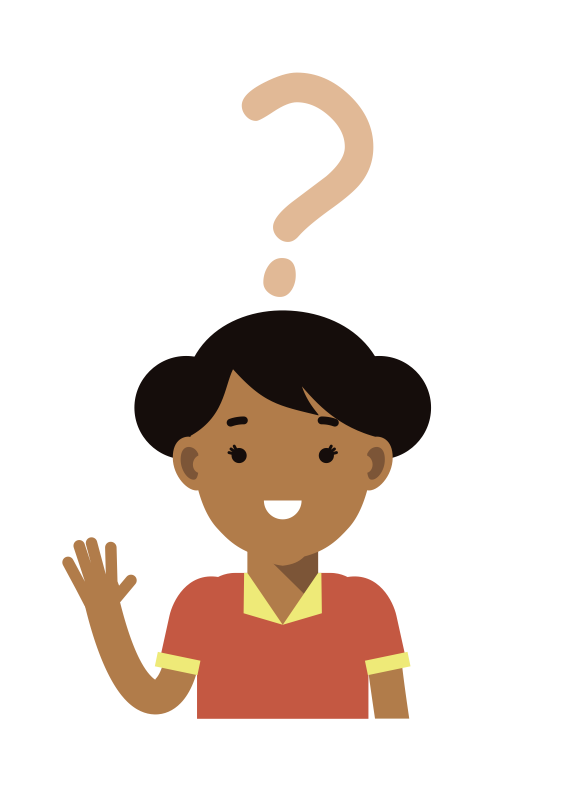  No, it is up to you to decide. If you don’t want to, it won’t change how you are looked after.  If you decide to take part but later on you change your mind, that’s ok. You can stop taking part at any time, without telling us why. Just tell your parents or guardian, the doctor or nurse that you want to stop.  If you don’t want to take part, we would be interested in talking to you and your family about this to find out more. |
| What will happen to me if I say yes? |
| 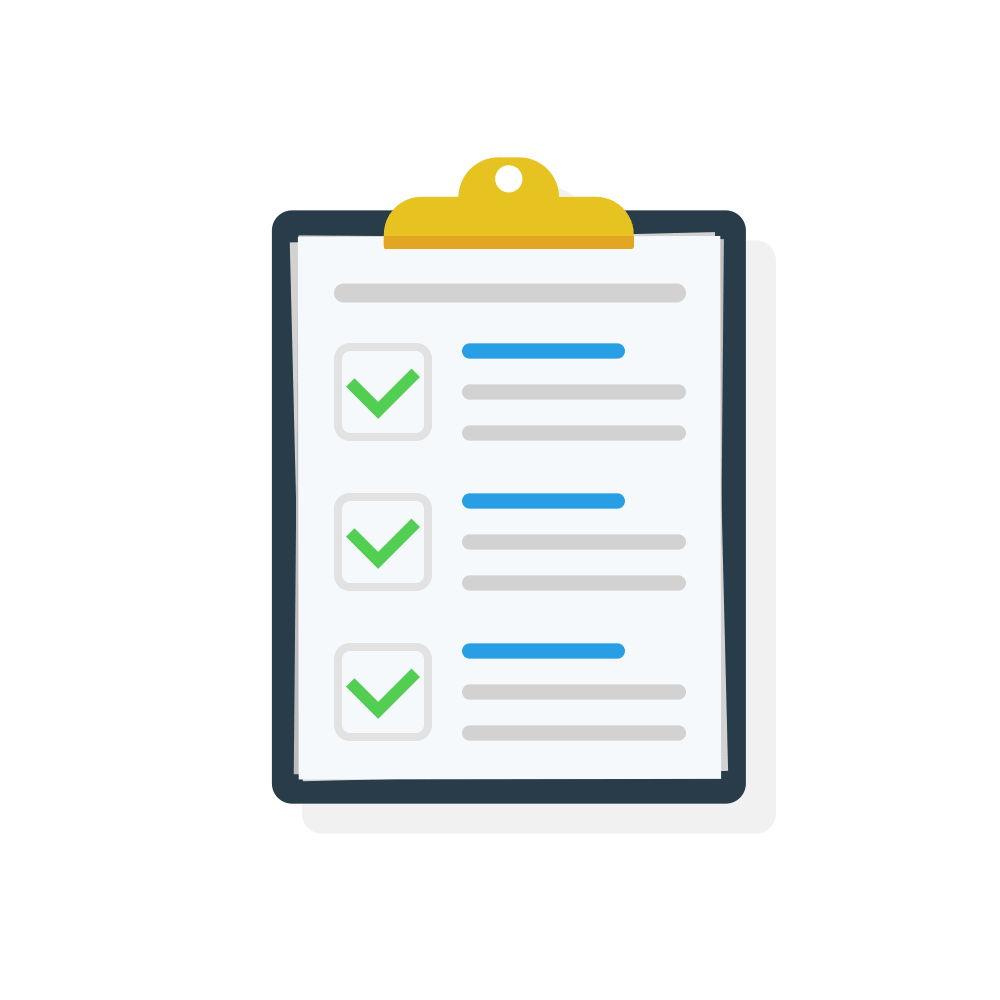You will be asked to write your name on the form at the end of this leaflet to say that you understand the study and what will happen and wish to take part. You will be given a copy of the form to keep, as well as this leaflet.  Your parents or guardian will need to sign a consent form to agree to you taking part.  There are two parts to the study. You can decide which you would like to participate in. You can take part in both.  **Part A – Main Study**   - You and your parents or guardian will be asked some **questions about your cavernoma** and about **how you are** **doing** by the study doctor or nurse. If this is done at the hospital, you will be asked if we may take a **small blood sample to look at your DNA for other studies**. If you don’t want to, that’s ok.   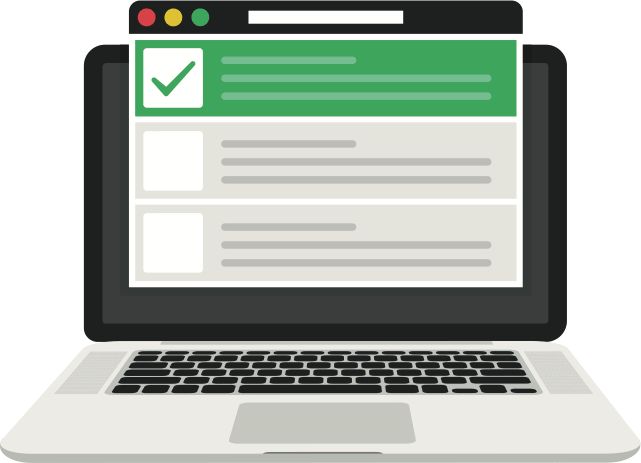   - You will then be put into **1 of 2 groups (**either **Treatment without surgery** or **Treatment including surgery)**. To make it work, nobody can pick which group you are put into. This will be **computer generated**. You will have the same chance of getting either of the two kinds of treatment. Everyone will be told straight away which group you have been put into. The study doctor will arrange for your treatment to be started. - 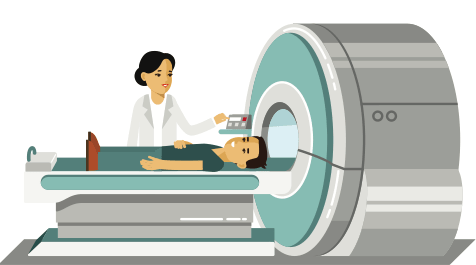You will be asked to come to the hospital with your parents or guardian after **6 months** to **answer some more questions about how you are doing** and do an **MRI** **scan of your brain** so the study doctor can look at your cavernoma. The scan will take around 20-30 minutes and you might find it noisy. You can say at any time if you want to stop the scan. If you didn’t give a **blood sample** before, you will be invited to give one. If you don’t want to, that’s ok. - After this, your parents or guardian will be contacted by the study doctor or nurse from time-to-time until the study is over to **answer some questions** and to **check how** **you are doing**.   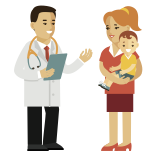   - After the study has finished, the study doctor will collect information about your health from your medical notes with your family’s permission.   **Part B - Information Study**   - You and your parents or guardian will also be invited to take part in an **interview** to discuss your experience of taking part in the study. - You and your parents or guardian were previously asked if it was ok for your **conversation** with them to be **audio-recorded**. We are interested in this conversation, and any others you and your family have about you joining the study. We will always ask for permission to **audio-record these conversations** too. |
| What are the possible benefits and possible risks of taking part? |
| We hope our research will benefit other children and adults with cavernoma. Doing this study will also help to improve how we do research in the future. There are risks associated with having any kind of treatment for brain cavernomas. The doctor will discuss these with you and your family.You might find having a blood sample taken uncomfortable and you might find the brain scanning machine noisy. Talk to your family and the study doctor or nurse if you are worried. Taking part in the study will take up some of your and your family’s time. |
| What if there are any problems? |
| Talk to your parents or guardian first or any of the research doctors and nurses. |
| Will my information be kept private? Will anyone know I’m taking part? |
| Your information will be kept confidential and in a safe place. Only people working on the study will be allowed to see it and only people involved with the study will know that you took part. |
| What will my blood sample be used for? |
| We will use your blood sample to look for genes in your DNA that that might affect how you might respond to medication. You won’t be given any results about this as part of this study. |
| What happens when the study is finished? |
| 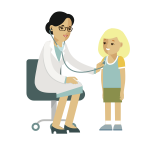  The doctor will continue to look after you as normal.  We will look at all the information we collected during the study and write a report about it. We will present the results to other researchers. Only people involved with the study will know that you took part. |
| Researcher Contact Details |
| If you have any further questions about the study please contact research team member [NAME] on [PHONE NUMBER] or email on: [EMAIL ADDRESS]. |
| **Thank you for reading this information and considering taking part. Please ask any questions you have.**  *Illustrations by Getty Images* |

**
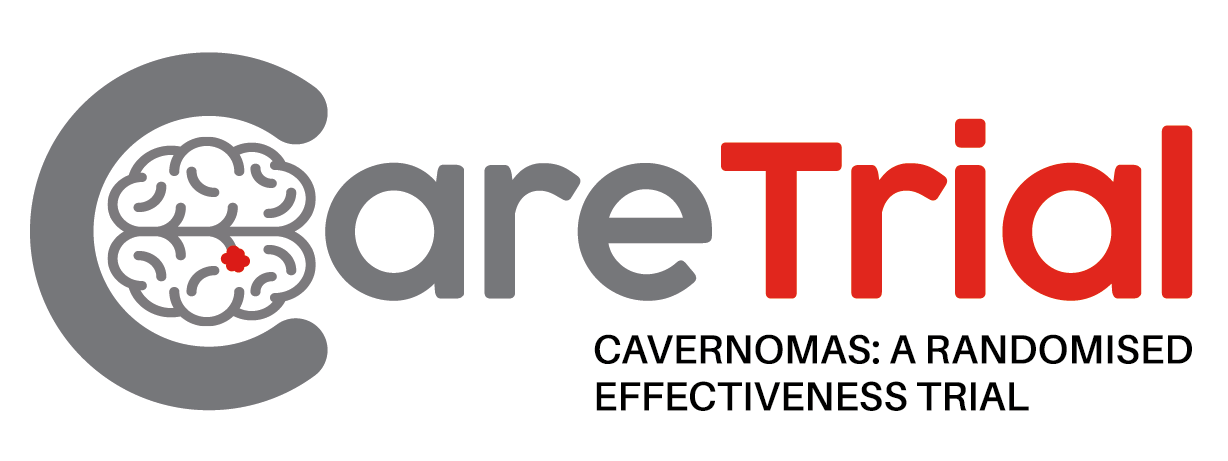
CARE Study**

**YOUNG PERSON’S ASSENT FORM (11-15 YEARS OLD)**

**(to be completed by the child and their parent/guardian)**

| Child (or if unable, parent/guardian on their behalf) to circle all they agree with: |  |
| --- | --- |
| Has somebody explained this study to you? | **Yes / No** |
| Do you understand what this study is about? | **Yes / No** |
| Have you asked all the questions you want? | **Yes / No** |
| Have your questions been answered in a way you understand? | **Yes / No** |
| Do you understand it’s OK to stop taking part at any time? | **Yes / No** |
| Are you happy to join in? | **Yes / No** |
| If any answers are ‘no’ or you **don’t** want to join in, **don’t** sign your name!  If you do want to take part, please write your name and today’s date |  |

|  | | |  |  |
| --- | --- | --- | --- | --- |
| Your name | | |  | Date |
| Your **parent or guardian** must write their name here too if they are happy for you to do the study | | | | |
| Print Name |  | Date |  | Signature |
| The **researcher** who explained this study to you needs to sign too: | | | | |
| Researcher |  | Date |  | Signature |

1x original – into Site File; 1x copy – to Participant; 1x copy – into medical record
