# Supplementary material for: Feasibility of comparing medical management and surgery (with neurosurgery or stereotactic radiosurgery) with medical management alone in people with symptomatic brain cavernoma – protocol for the Cavernomas: A Randomised Effectiveness (CARE) pilot trial
Source: BMJ Open. 2023 Aug 9;13(8):e075187. doi: 10.1136/bmjopen-2023-075187 (PMC10414059; doi:10.1136/bmjopen-2023-075187)
Supplement: Supplementary data [file bmjopen-2023-075187supp003.zip › 02 PIL & CF/CARE - Information Study Adult Patient Consent Form V2.0 22Mar2021 Clean.docx]

**CARE Trial (Information Study)**

**Adult Patient Informed Consent Form**

| *Please ask the patient to initial against each statement to indicate their agreement:* | | ***Patient to initial*** | | |
| --- | --- | --- | --- | --- |
|  |  | |  |  |
| 1. | I have read and understood the Patient Information Leaflet  (dated ____/____/____, version ____). I have had the opportunity to ask questions about the study and received satisfactory answers to my questions. | |  |  |
|  |  |  |  |  |
|  |  | |  |  |
| 2. | I understand that I am free to withdraw from the information study at any time without giving a reason and that withdrawing from the study will not affect my medical care or legal rights. | |  |  |
|  |  |  |  |  |
|  |  | |  |  |
| 3. | I agree to the recording of consultations and telephone discussions between me and the doctors, nurses and other healthcare staff involved in my care. I agree to my recorded data from my recorded consultations/discussions being transferred to the University of Bristol. Data will be de-identified so that I cannot be identified from any data used and I understand that my de-identified data may be used for analysis and training in the CARE study and for teaching and research purposes, now and in the future.  *Site team to mark NA if not applicable* | | Yes No | |
| 4. | I agree to my contact details being sent to a researcher at the University of Bristol so I can be contacted about the possibility of an interview. | | Yes No | |
|  |  | |  |  |
|  |  | |  |  |
| 5.. | I understand that after the study the data collected for the Information Study will be made “controlled data”. I understand that this means the de-identified data will be available to other researchers who secure the necessary approvals. I understand that this means that data may be used for purposes not related to this study, but it will not be possible to identify me from these data. | | Yes No | |
|  |  | |  |  |

_________________________ ___________________________ __________________

Name of patient Signature Date

_________________________ ___________________________ ____________________

Name of person taking consent Signature Date

1 copy for patient; 1 (original) for research team; 1 copy to be kept with paper hospital notes or stored electronically
