# Supplementary material for: Feasibility of comparing medical management and surgery (with neurosurgery or stereotactic radiosurgery) with medical management alone in people with symptomatic brain cavernoma – protocol for the Cavernomas: A Randomised Effectiveness (CARE) pilot trial
Source: BMJ Open. 2023 Aug 9;13(8):e075187. doi: 10.1136/bmjopen-2023-075187 (PMC10414059; doi:10.1136/bmjopen-2023-075187)
Supplement: Supplementary data [file bmjopen-2023-075187supp003.zip › 02 PIL & CF/CARE - Short PIL (Parent Guardian) V1.0 28Jan2021.docx]

**C**avernomas: **A** **R**andomised **E**ffectiveness (**CARE**) Trial

# Short Information Leaflet for Parents/Guardians of Paediatric Participants

## We are asking if your child would join the CARE Trial

To help you decide whether your child should take part in this research study, it’s important that you understand why the research is being done and what it will involve. A member of our team (a doctor or nurse involved in the study at your hospital) will go through this information leaflet with you. Please take time to read it carefully. Talk to others if you wish. Contact us if there’s anything that’s not clear, or if you would like more information. Take time to decide whether you wish your child to participate.

## Why has your child been invited to take part?

Your child has been asked to take part for two reasons:

- First, your child had symptoms caused by a brain cavernoma.
- Second, their doctor isn’t sure whether they should have:
  - **‘Treatment without surgery’** for their symptoms, or
  - **‘Treatment including surgery’** for their symptoms and either:
    - trying to remove the brain cavernoma with **neurosurgery**, or
    - trying to stabilise the brain cavernoma with focussed radiation treatment (known as **stereotactic radiosurgery**)

## Do they have to take part?

No, it’s up to you to decide whether your child should take part in each of the two parts of the study. Your decision will not affect their standard medical care. If you do not want your child to take part, we are interested to interview you to understand why. Your feedback will help us design the CARE Trial and future studies. Let the research team know if you are happy to be interviewed.

## Background

A cavernoma is a cluster of blood vessels that form blood-filled ‘caverns’ that look like a raspberry. Brain cavernomas can cause strokes or epileptic seizures. The problems caused by a stroke from a cavernoma depend on its location in the brain. These symptoms are usually mild, but sometimes they cause disability, other health or social problems or, very rarely, death.

Standard **‘treatment without surgery’** for most people with brain cavernoma involves treating their symptoms, preventing seizures with drugs, or helping recovery with rehabilitation.

About one fifth of people in the UK with brain cavernoma have ‘**treatment including surgery**’, involving standard treatment and either **neurosurgery** or **stereotactic radiosurgery**. You should have been informed about these treatments by your consultant. More information about the risks and benefits is available in the supplementary information leaflet.

## Why do we need to undertake this research?

The risks and benefits of treatment without surgery seem similar to treatment including surgery. There is no reliable evidence about which is better. This can leave doctors and patients uncertain about whether to use surgery. Answering this uncertainty is the top priority for research into cavernoma.

This pilot study has two parts:

- The Randomised Study is comparing treatment without surgery to treatment including surgery for people of all ages with symptoms from brain cavernoma.
- The Information Study looks at how information is given to patients, and what patients think about taking part.

These two parts of the CARE Trial will tell us if enough eligible patients take part to indicate that a larger trial could be done to resolve this uncertainty.

## What will happen if your child takes part?

The diagram below summarises what taking part involves:


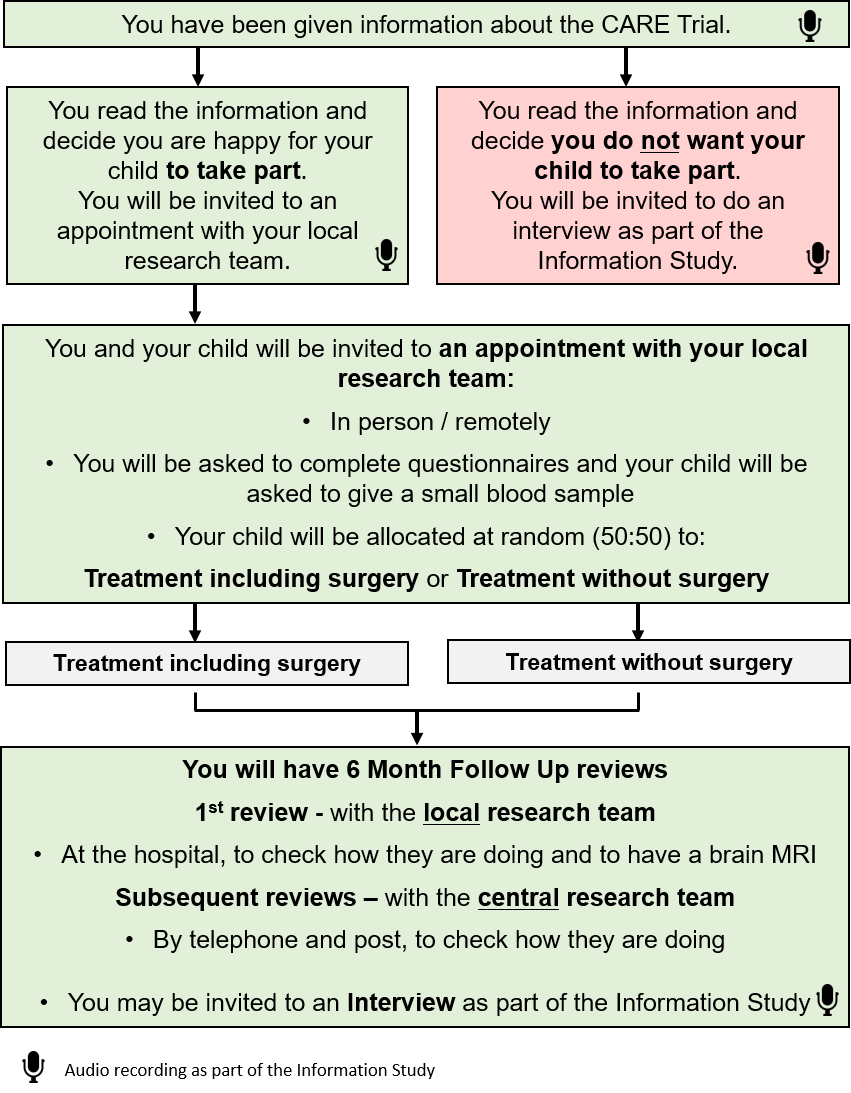


We hope that this research study will help you and others diagnosed with symptoms due to brain cavernoma.

## What happens now?

If you are interested in learning more about the CARE Trial, you can:

- Read the **Supplementary Patient Information Leaflet**, which the research team will provide.
- Talk to **your** **child’s** **hospital doctor.**
- Contact and talk to Cavernoma Alliance UK:

TO BE ADDED BEFORE USE AT SITES:

[NAME]

[EMAIL ADDRESS]

[PHONE NUMBER]

- - Via their website: [www.cavernoma.org.uk](http://www.cavernoma.org.uk)

## If you decide to take part

Let your hospital doctor giving you care for your brain cavernoma know and they will help you get involved.

**Thank you for reading this patient information leaflet and considering taking part in this research study which will potentially help your child and others diagnosed with cavernoma.**
