# Supplementary material for: Feasibility of comparing medical management and surgery (with neurosurgery or stereotactic radiosurgery) with medical management alone in people with symptomatic brain cavernoma – protocol for the Cavernomas: A Randomised Effectiveness (CARE) pilot trial
Source: BMJ Open. 2023 Aug 9;13(8):e075187. doi: 10.1136/bmjopen-2023-075187 (PMC10414059; doi:10.1136/bmjopen-2023-075187)
Supplement: Supplementary data [file bmjopen-2023-075187supp003.zip › 02 PIL & CF/CARE - Childrens 6-10 years PIL AF V1.0 08Dec2020.docx]

**Information Leaflet and Assent Form for children (6-10 years old)**

We would like you and your mum or dad or the grown up looking after you to help us with our research study.

We will explain why the study is being done and what it will involve. If something isn’t clear or if you have more questions, you can ask your mum or dad or grown up that looks after you.

Thank you for reading this**.**


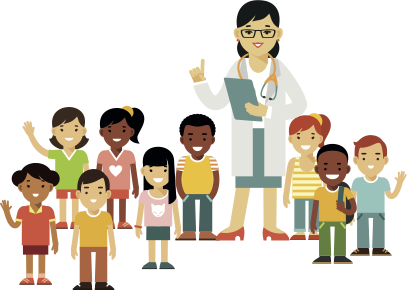


| What is a research study? Why is this being done? |
| --- |
| A research study is something you do when you want to learn about something and answer questions.  We want to find out which is the best way to look after people with brain cavernomas. A cavernoma is made up of abnormal blood vessels and can be found in the brain and/or spinal cord and looks like a raspberry.  There are 2 kinds of treatment used by doctors:   1. **Treatment without surgery.** This may involve taking medicines or other treatments such as exercises and activities that will help you feel better if you’ve been poorly 2. **Treatment including surgery.** This involves surgery to remove the cavernoma (neurosurgery) or a kind of surgery using radiation (radiosurgery) to stabilise the cavernoma and may also include taking medicines or other treatments.   Doctors don’t always know which one is best. The way to find out is to do research. |
| Why have I been chosen? |
| We asked you because you have a brain cavernoma. |
| Do I have to help? |
| 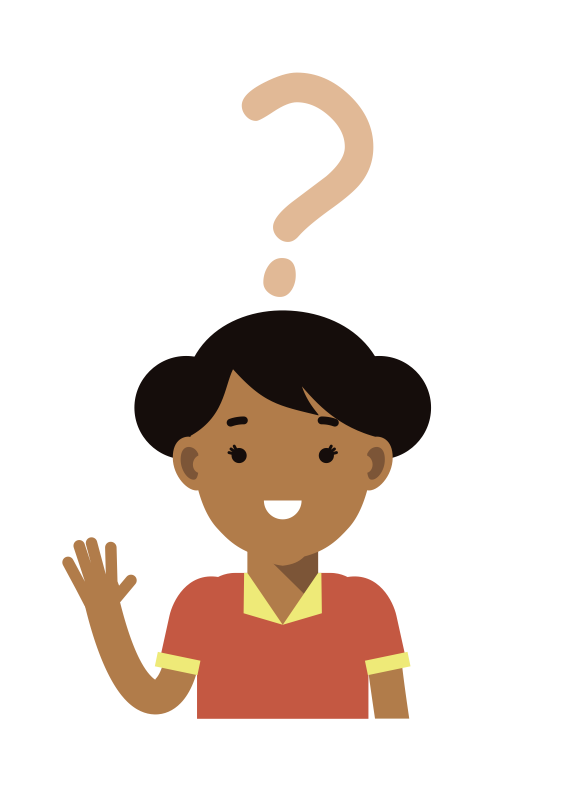  You can help if you want to. If you don’t want to, that’s okay too, nobody will mind. You can tell us why you didn’t want to help if you like. |
| What will happen to me if I say yes? |
| 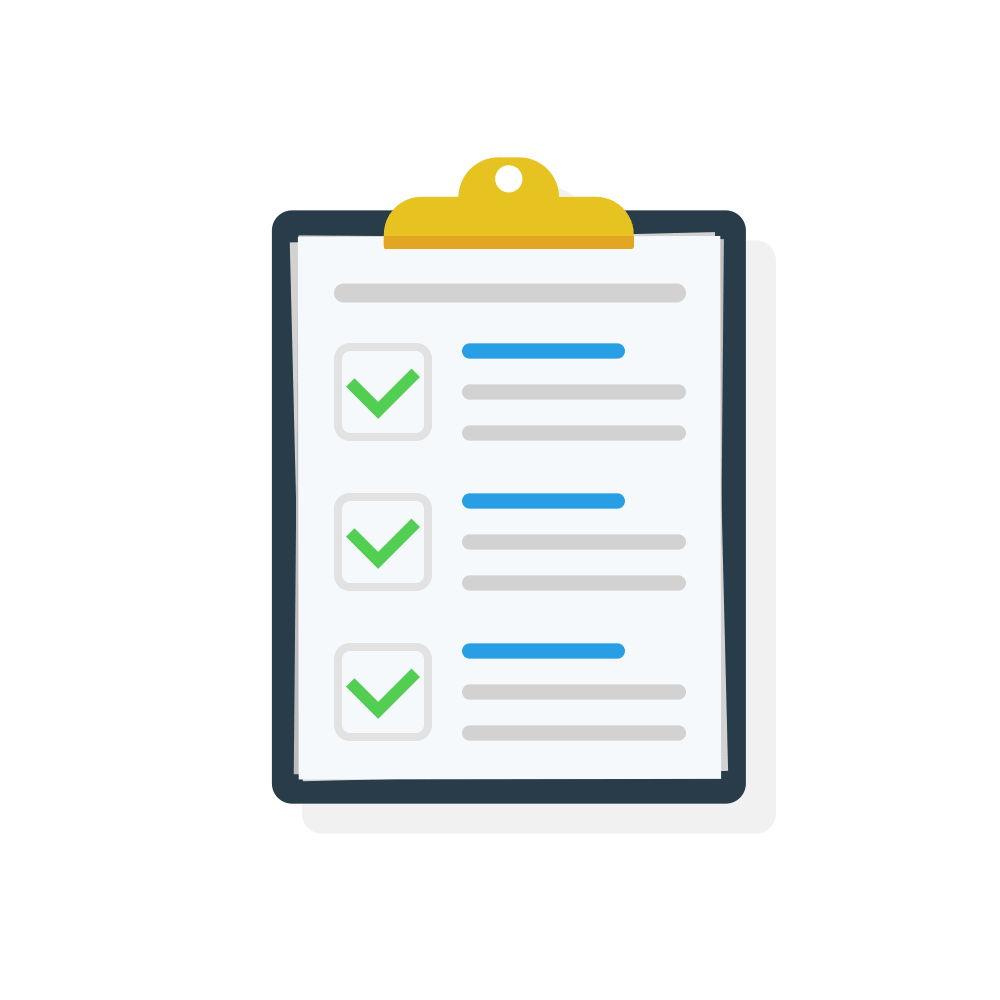  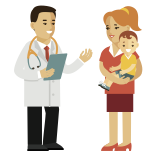If you are able, we will ask you to write your name on a form to say that you understand the study and what will happen. You will be given your own form to keep, as well as this leaflet. Your mum or dad or the grown up looking after you and the doctors and nurses will take care of everything else.  The study doctor will record the conversations you and your family have with them about the study. The study doctor or nurse will collect some information from you and your mum or dad or grown up looking after you. We will ask you if we may take a small blood sample. If you don’t want to, that’s ok.  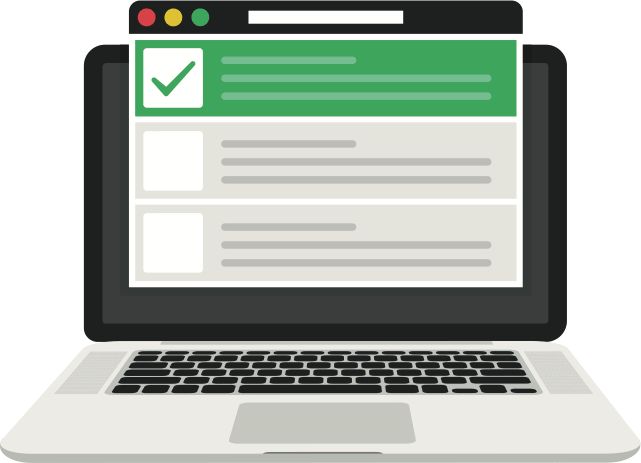  You will then be put into 1 of 2 groups:   1. One group will get treatment without surgery 2. One group will get treatment including surgery   To make it work, nobody can pick which group they are in. This will be decided by a computer. The study doctor will tell you which group you are in.  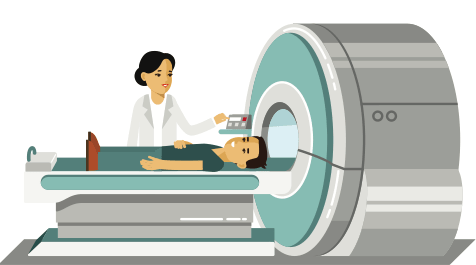  You and your mum or dad or grown up looking after you will come to the hospital 6 months later to see how you are doing. You will have a brain scan to look at your cavernoma. The machine is quite noisy. If you are worried, you can tell your mum or dad or the grown up looking after you, or the doctor or nurse.  After this, the study doctor or nurse will contact your mum or dad or the grown up looking after you twice a year to answer some more questions and to check how you are doing.  The study doctor will also collect information about how you are doing after the study is over if your mum or dad or the grown up looking after you say it’s ok. |
| Will anything good or bad happen to me if I take part? |
| Nothing bad will happen to you for choosing to help us. If you are worried, tell your family or the doctor or nurse.  We hope the study will help other people with cavernoma by trying to find out which treatment is best for them.  If you enjoyed helping with this study, you can tell us later on. |
| What do I have to do now? |
| If you want to help, your mum or dad or the grown up looking after you will need to say it’s okay. |
| What if I have questions? |
| 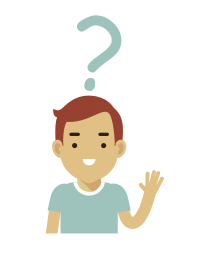  If you have any questions, you can ask your mum or dad or the grown up looking after you. You can also ask the doctor or nurse in the hospital. |
| **Thank you for reading this information and thinking about taking part. Please ask any questions you have.**  *Illustrations by Getty Images* |

**CARE Study**

**CHILDREN’S ASSENT FORM (6-10 YEARS OLD)**

**(to be completed by the child and their parent/guardian)**

| Child (or if unable, parent/guardian on their behalf) to circle all they agree with: |  |
| --- | --- |
| Has somebody explained this study to you? | **Yes / No** |
| Do you understand what this study is about? | **Yes / No** |
| Have you asked all the questions you want? | **Yes / No** |
| Have your questions been answered in a way you understand? | **Yes / No** |
| Do you understand it’s OK to stop taking part at any time? | **Yes / No** |
| Are you happy to join in? | **Yes / No** |
| If any answers are ‘no’ or you **don’t** want to join in, **don’t** sign your name!  If you do want to take part, please write your name and today’s date |  |

|  | | |  |  |
| --- | --- | --- | --- | --- |
| Your name | | |  | Date |
| Your **parent or guardian** must write their name here too if they are happy for you to do the study: | | | | |
| Print Name |  | Date |  | Signature |
| The **researcher** who explained this study to you needs to sign too: | | | | |
| Researcher |  | Date |  | Signature |

1x original – into Site File; 1x copy – to Participant; 1x copy – into medical record
