# Supplementary material for: Feasibility of comparing medical management and surgery (with neurosurgery or stereotactic radiosurgery) with medical management alone in people with symptomatic brain cavernoma – protocol for the Cavernomas: A Randomised Effectiveness (CARE) pilot trial
Source: BMJ Open. 2023 Aug 9;13(8):e075187. doi: 10.1136/bmjopen-2023-075187 (PMC10414059; doi:10.1136/bmjopen-2023-075187)
Supplement: Supplementary data [file bmjopen-2023-075187supp003.zip › 02 PIL & CF/CARE - Consent Form (Child With Capacity) - Scotland V2.0 (22Mar2021) Clean.docx]

**CARE Trial (Randomised Study)**

**Child with Capacity Patient Informed Consent Form (Scotland)**

| Please **initial** box | |
| --- | --- |
| 1. I confirm that I have read and understand the information sheet (Version Number:___, Date:____________) for the CARE pilot trial. I have had the opportunity to consider the information, ask questions and have had these questions answered satisfactorily. |  |
| 1. I understand that my participation is voluntary and that I am free to withdraw at any time, without giving any reason and without my medical care and/or legal rights being affected. |  |
| 1. I give permission for the research team to access my medical records for the purposes of this research study. |  |
| 1. I understand that relevant sections of my medical notes and data collected during the study may be looked at by individuals from the Sponsor (University of Edinburgh and/or NHS Lothian), from regulatory authorities or from the NHS organisation where it is relevant to my taking part in this research. I give permission for these individuals to have access to my data and/or medical records. |  |
| 1. I give permission for my personal information (including name, address, email address, date of birth and telephone number) to be entered into an electronic database managed by the Edinburgh Clinical Trials Unit and that relevant staff will have access to this information for administration purposes. I understand that my contact details will be used to contact me about the study. |  |
| 1. I give permission for my NHS or Community Health Index (CHI) number, and hospital number to be collected and passed to the Edinburgh Clinical Trials Unit. |  |
| 1. I agree to my General Practitioner being informed of my participation in the study and providing follow-up information about my current and future health problems for the lifetime of the study. |  |
| 1. I understand that data collected about me during the study may be converted to anonymised data. |  |
| 1. I agree that the researchers can find out how I am getting on by contacting me every 6 months for the duration of the study follow up period. |  |
| 1. My relatives, carers, or close personal contacts named on the Contact Form are willing to provide information about how I am getting on. These people or others caring for me may provide this information if I cannot be contacted or I am no longer able to make decisions for myself. |  |
| 1. I agree to give a blood sample which will be used for genetic DNA analysis. | Yes No |
| 1. If my DNA sample is analysed in the future, I would like to be informed about any results that are relevant to my health. | Yes No |
| 1. I agree to the following being shared with other researchers and being used in future studies, whatever happens to me:  - Anonymised data - Anonymised brain imaging - Anonymised blood or DNA sample | Yes No  Yes No  Yes No |
| 1. I understand that the information held and maintained by NHS Digital and other central UK NHS bodies may be used to help contact me or provide information about my health and use of services. I agree to my NHS or Community Health Index (CHI) number being used to gather information relevant to my participation after the study has finished. | Yes No |
| 1. I would like to be informed of the results of the CARE pilot trial. | Yes No |
| 1. I agree to my contact details being kept on record and am happy to be contacted about similar research in the future. | Yes No |
| 1. I agree to take part in the above study. |  |

|  |  |  |  |  |
| --- | --- | --- | --- | --- |
| Name of Person Giving Consent |  | Date |  | Signature |
|  |  |  |  |  |
| Witness (if participant has provided verbal consent) |  | Date |  | Signature |
|  |  |  |  |  |
| Name of Person Receiving Consent (researcher) |  | Date |  | Signature |

1x original – into Site File; 1x copy – to Participant; 1x copy – into medical records
