# Supplementary material for: Feasibility of comparing medical management and surgery (with neurosurgery or stereotactic radiosurgery) with medical management alone in people with symptomatic brain cavernoma – protocol for the Cavernomas: A Randomised Effectiveness (CARE) pilot trial
Source: BMJ Open. 2023 Aug 9;13(8):e075187. doi: 10.1136/bmjopen-2023-075187 (PMC10414059; doi:10.1136/bmjopen-2023-075187)
Supplement: Supplementary data [file bmjopen-2023-075187supp003.zip › 02 PIL & CF/CARE - Information Study Health Care Professional PIS and CF V2.0 22Mar2021 Clean.docx]

**Health and Research Professionals’ Information and Consent Form**

***Recording of consultations and/or interviews***

The CARE pilot randomised controlled trial (RCT) has an integrated Qualitative Recruitment Intervention (QRI – referred to as the *Information Study* in patient facing documents). The QRI has been set up to understand how patients eligible for CARE are identified and invited to join the study. This information leaflet explains the purpose and conduct of the QRI to enable you to make an informed decision about participation. Please ask a member of the qualitative research team if there is anything that is not clear or if you would like more information. You can find contact details at the end of this leaflet.

**What is the purpose of this study?**

The QRI aims to optimise recruitment to CARE and facilitate clear and balanced information provision about treatment options to patients. To do this, we would like to record appointments where CARE is discussed with patients, interview professionals, and observe CARE study meetings to help us understand how recruitment operates in different centres.

**Why have I been chosen?**

You have been chosen because of your involvement in the CARE study. Your role may encompass discussing treatment options with patients, recruiting eligible patients, or you may have responsibility for trial conduct and oversight.

**What do I have to do if I take part?**

Taking part in this study will involve one or more of the following:

1. **Recording your recruitment discussions:** we will ask you to record discussions you have with patients about participating in the CARE study (using a digitally encrypted audio-recorder or using the secure recording facility available within the video conferencing software used).
2. **Being interviewed**: we may invite you to attend one or more interviews with a qualitative researcher to discuss your views on the CARE study. This will be arranged at a time and place that is convenient to you and should last for approximately 30-60 minutes. The interview will be recorded (with your consent) so that the researchers can listen to the recording and make a transcript of the discussion. Interviews may be carried out over the telephone or via MS Teams and may be captured initially as an audio or video recording, depending on the platform used. Only the audio-recording will be transferred to the University of Bristol for data analysis.
3. A researcher may observe and record CARE study meetings if consent has been obtained from all members in attendance.
4. You may be invited to attend individual and group training sessions, based on the qualitative research findings. This training will be supportive, with the aim of sharing best practice and using your experience to devise solutions to recruitment challenges.

You will be asked to sign a consent form that includes separate clauses for each of the above activities. Any activities that include patients/family members will only be carried out if consent has been obtained from all parties present. This information will be presented in separate leaflets for patients and their families.

**Do I have to take part?**

It is entirely up to you to decide whether to take part in this study. If you do decide to take part, you will be given this information sheet to keep and asked to sign a consent form. If you decide to take part you are free to withdraw from the study or any of its components at any time, without giving a reason. You are also free to refuse to answer any specific interview question or withdraw from an interview or training session without giving a reason.

**Will my taking part in this study be kept confidential?**

All information collected about you during this research will be kept strictly confidential. If you are interviewed, we will not inform anyone that you have taken part. Audio-recorded data will be transferred to the University of Bristol to be used for research and training**.** All recordings will be labelled with a reference number (not with your name) to hide your identity. Interviews and some of the recorded appointments will be transcribed by a University of Bristol employee or University of Bristol approved transcribing service. All transcripts will also be de-identified and stored securely. All individual feedback will be treated in confidence.

We may wish to use quotes and play parts of your audio-recordings (from interviews and appointments/meetings) as part of publications, teaching and presentations at academic meetings. If we do use any of your data, all quotes will be de-identified (and voices modified if necessary) so that you cannot be recognised from any of the information we present. We may also use the data collected (including quotes) in our future research, teaching and publications looking at common issues across studies. You will not be identified in any way, in any presentation, report or publication. Please indicate on the consent form if you are happy for us to use your recordings in the above ways.

**What are the possible disadvantages and risks of taking part?**

There are no physical risks to taking part. You will have to set aside up to 45 minutes of your time to take part in each interview. You may be requested to take part in more than one interview over the course of the CARE study, though these will be arranged at your convenience. Taking part in one interview does not mean you are under any obligation to agree to a follow up interview.

**What are the possible benefits of taking part?**

If you are involved in recruitment of participants, you may receive individual support in your role as a recruiter and will have opportunities to discuss any difficulties you may be experiencing with the recruitment process. You may receive individually tailored feedback which will enable you to continue to improve your communications skills. Some people find that taking part in interviews enables them to talk through their views and experiences, which can help them to reflect on their practice.

**What will happen if I don’t want to carry on with this study?**

You are free to withdraw from the study or any of its components at any point. If you withdraw, any information collected before your withdrawal will be kept and used for the research, unless you specify that you would like this to be destroyed.

**What if something goes wrong?**

It is extremely unlikely that you will be harmed by taking part. If you do experience any difficulties with taking part, please discuss this with the qualitative researchers so that we can try to resolve the matter.

**What will happen to the results of the research study?**

After a thorough review by independent experts, a report of the study findings and academic papers will be published in respected journals. Findings may also be presented at academic conferences/workshops or used for training purposes. You will not be identified in any report, publication or presentation.

**What will happen to my data?**

At the end of the study, any transcriptions made of your recordings will be made “Controlled Access”. This means that transcripts will be stored in an online database, which can be accessed by approved individuals who are interested in conducting their own analyses of the data. These individuals will have to apply for permission to do this, and applications will be assessed by an independent committee. We will therefore have no control over how these data are used in the future. However, all data will be de-identified before they are made available, and there will be no way to identify you or any other individuals mentioned in your interviews/appointments. Sharing access of research data and findings is considered good research practice and is a requirement of many funding bodies and scientific journals. Sharing data helps to maximise the impact of money invested into conducting research studies and can encourage new avenues of research.

**Who is organising and funding the research?**

The study is funded by the National Institute of Health Research (funding reference 128694) and is led by Prof Rustam Al-Shahi Salman at the University of Edinburgh. The study is sponsored by the University of Edinburgh and NHS Lothian and is managed by Edinburgh Clinical Trials Unit.

**Who has reviewed the study?**

This study has been reviewed and given a favourable opinion by the Yorkshire & The Humber - Leeds East Research Ethics Committee (21/YH/0046).

**Who do I contact if I want further information or have concerns?**

If you have any concerns or queries, you are welcome to contact the qualitative researchers below:

**Dr Julia Wade**

[**Julia.wade@bristol.ac.uk**](mailto:Julia.wade@bristol.ac.uk)

**07847 618455**

**Health and Research Professionals’ Consent Form**

***Audio-recording of consultations and/or interviews***

| ***This form should be completed by the member of staff.*** | | ***Please initial each box:*** | |
| --- | --- | --- | --- |
|  |  |  | |
| 1. | I confirm that I have received enough information about this research and have  had the opportunity to ask questions. These questions have been answered clearly and satisfactorily. |  |  |
|  |  |  | |
|  |  |  | |
| 2. | I understand that I am free to withdraw from the study at any time without giving a reason and that withdrawing from the study will not affect my legal rights. I agree to any information collected before my withdrawal being retained and used for this research. |  |  |
|  |  |  | |
|  |  |  | |
| 3. | I agree to the audio-recording of consultations with patients during which the CARE Study is discussed. I agree to my recorded data from my recorded consultations being transferred to the University of Bristol for analysis for the CARE study. | Yes No | |
|  |  |  |  |
| 4. | I agree to take part in an audio-recorded interview investigating my experiences of recruiting patients to the CARE study. | Yes No | |
| 5. | I agree for my data to be retained for training, teaching and research purposes, now and in the future. | Yes No | |
| 6. | I agree for my transcriptions to be made “Controlled Access”. | Yes No | |
|  |  |  |  |
| 7. | I agree to take part in this study, which involves recording consultations and / or interviews. |  |  |
|  |  |  |  |

_________________________ ___________________________ __________________

Name of participant Signature Date

_________________________ ___________________________ ____________________

Name of person taking consent Signature Date

1 copy for health professional; 1 for research team (original)
