# Supplementary material for: Feasibility of comparing medical management and surgery (with neurosurgery or stereotactic radiosurgery) with medical management alone in people with symptomatic brain cavernoma – protocol for the Cavernomas: A Randomised Effectiveness (CARE) pilot trial
Source: BMJ Open. 2023 Aug 9;13(8):e075187. doi: 10.1136/bmjopen-2023-075187 (PMC10414059; doi:10.1136/bmjopen-2023-075187)
Supplement: Supplementary data [file bmjopen-2023-075187supp003.zip › 02 PIL & CF/CARE - Information Study Parent Guardian Consent Form V2.0 22Mar2021 Clean.docx]

**CARE Trial (Information Study)**

**Parent/Guardian Informed Consent Form**

| *Please ask the parent/guardian to initial against each statement to indicate their agreement:* | | ***Parent/guardian to initial*** | | |
| --- | --- | --- | --- | --- |
|  |  | |  |  |
| 1. | I have read and understood the Patient Information Leaflet  (dated ____/____/____, version ____). I have had the opportunity to ask questions about the study and received satisfactory answers to my questions. | |  |  |
|  |  |  |  |  |
|  |  | |  |  |
| 2. | I understand that I am free to withdraw from the information study at any time without giving a reason and that withdrawing from the study will not affect my child’s medical care or legal rights. | |  |  |
|  |  |  |  |  |
|  |  | |  |  |
| 3. | I agree to the recording of consultations and telephone discussions between me and my child and the doctors, nurses and other healthcare staff involved in my child’s care. I agree to the recorded data from these recorded consultations/discussions being transferred to the University of Bristol. Data will be de-identified so that my child and I cannot be identified from any data used and I understand that this de-identified data may be used for analysis and training in the CARE study and for teaching and research purposes, now and in the future.  *Site team to mark NA if not applicable* | | Yes No | |
| 4. | I agree to my contact details being sent to a researcher at the University of Bristol so I can be contacted about the possibility of an interview. | | Yes No | |
|  |  | |  |  |
| 5. | I understand that after the study, the data collected for the Information Study will be made “controlled data”. I understand that this means the de-identified data will be available to other researchers who secure the necessary approvals. I understand that this means that data may be used for purposes not related to this study, but it will not be possible to identify me or my child from these data. | | Yes No | |
|  |  | |  |  |

________________________ ___________________________ ____________________

Name of parent/guardian Signature Date

________________________ ___________________________ ____________________

Name of person taking consent Signature Date

1 copy for patient; 1 (original) for research team; 1 copy to be kept with paper hospital notes or stored electronically
