# Supplementary material for: Feasibility of comparing medical management and surgery (with neurosurgery or stereotactic radiosurgery) with medical management alone in people with symptomatic brain cavernoma – protocol for the Cavernomas: A Randomised Effectiveness (CARE) pilot trial
Source: BMJ Open. 2023 Aug 9;13(8):e075187. doi: 10.1136/bmjopen-2023-075187 (PMC10414059; doi:10.1136/bmjopen-2023-075187)
Supplement: Supplementary data [file bmjopen-2023-075187supp004.pdf]

Statistical Analysis Plan	CARE
Version No	1.0
Date Finalised	8th Dec 2022

Cavernomas A Randomised Effectiveness (CARE) pilot trial, to address the effectiveness of active treatment (with neurosurgery or stereotactic radiosurgery) versus conservative management in people with symptomatic brain cavernoma

Statistical Analysis Plan

CONFIDENTIAL

**Version No**	v1.0
**Date Finalised**	8 Dec 2022
**Author(s)**	Dr Jacqueline Stephen
**CI Name**	Prof Rustam Al-Shahi Salman
**CI Email address**	Rustam.Al-Shahi@ed.ac.uk

Signatures	
**Trial Statistician:**	**Date: 12 Dec 2022**
**Chief Investigator:** **RUSTAM AL-SHAHI SALMAN**	**Date:** 12 December 2022

**Document Control**		
**Version No**	**Date**	**Summary of Revisions**
1.0	8 Dec 2022	Initial Creation

Loan JJM, *et al. BMJ Open* 2023; 13:e075187. doi: 10.1136/bmjopen-2023-075187

Statistical Analysis Plan	CARE
Version No	1.0
Date Finalised	8th Dec 2022

Table of Contents

List of Abbreviations 3
1. Introduction 4
2. Statistical Methods section from the protocol 4
3. Overall Statistical Principles 5
4. List of Analyses 5
4.1 Outcomes 5
4.2 Serious adverse events 9
5. Validation and QC 10
6. Data sharing 10
7. References 10

Statistical Analysis Plan	CARE
Version No	1.0
Date Finalised	8th Dec 2022

List of Abbreviations

Abbreviation	Full name
CI	Confidence interval
CRF	Case report form
ECTU	Edinburgh Clinical Trials Unit
FND	Focal neurological deficit
ICH	Intracranial haemorrhage
IQR	Inter quartile range
ITT	Intention-to-treat
mRS	Modified rankin scale
RCT	Randomised controlled trial
SAE	Serious adverse events
SD	Standard deviation
SOP	Standard operating procedure

Loan JJM, *et al. BMJ Open* 2023; 13:e075187. doi: 10.1136/bmjopen-2023-075187

Statistical Analysis Plan	CARE
Version No	1.0
Date Finalised	8th Dec 2022

1. Introduction

This document details the criteria to be used for the definition of the analysis populations and the statistical methodology for analysis of CARE, a two-arm, parallel group randomised feasibility trial which aims to estimate the feasibility of performing a definitive main phase randomised controlled trial (RCT) comparing medical management to medical and surgical management (with neurosurgery or Gamma Knife stereotactic radiosurgery, according to their availability in clinical practice) for improving outcomes for people with symptomatic brain cavernoma.

The aim is to randomise approximately 60 participants to groups in a 1:1 ratio, to medical management alone, or medical and surgical management, stratified by preferred type of surgical management. If there is no clear preference for the type of surgical management, and both are available, the patient will be randomly allocated to either neurosurgery or stereotactic radiosurgery, and then randomised between medical management alone, or medical and surgical management.

This document has been compiled according to the Edinburgh Clinical Trials Unit (ECTU) standard operating procedure (SOP) "Statistical Analysis Plans v6.0" and has been written based on information contained in the study protocol version 2.0, dated 22nd March 2021.

The pilot phase of CARE will be submitted for publication and reported according to the CONSORT 2010 extension to randomised pilot and feasibility trials.[1]

2. Statistical Methods section from the protocol

In this pilot phase, analyses are descriptive only, and there will be no formal statistical tests.

We will quantify the number and proportions (with 95% confidence intervals to reflect their precision) of patients who are screened, eligible, approached, consent and are randomised. We will construct a CONSORT diagram to summarise the distribution and progress of participants in the trial including the numbers of withdrawals.[1]

We will report descriptively the following: the number and the proportion of the collaborating sites that take part and recruit participants to the CARE pilot trial; research teams' implementation of trial procedures measured by number and type of protocol deviation; the numbers of participants allocated to neurosurgery and stereotactic radiosurgery; adherence to the allocated intervention; completeness of follow-up that would be due at each 6-month interval; completeness of baseline, imaging and outcome data; the frequency of outcome events overall and in an intention-to-treat analysis keeping patients in the treatment group to which they were allocated during all available follow-up.

We will also compare descriptively the characteristics of eligible patients who are screened and do not participate in the CARE pilot trial to eligible patients who are randomised using the

Loan JJM, *et al. BMJ Open* 2023; 13:e075187. doi: 10.1136/bmjopen-2023-075187

characteristics recorded on the screening logs to assess generalisability (external validity) and any recruitment bias.

We will assess measures of functional outcome, to assess which has suitable statistical properties for use in a main phase trial (such as lack of floor/ceiling effects). We will assess whether such a measure (like the method we have used before[2]) would be more suitable as a primary outcome in place of intracranial haemorrhage.

3. Overall Statistical Principles

The analysis dataset for the trial will include all screened patients in addition to eligible, approached, consented, and randomised participants.

All analyses will be based on the intention to treat (ITT) principle with patients analysed according to allocated treatment, irrespective of whether they adhered to the allocated treatment, in the group to which they were allocated.

In general terms, categorical data will be presented using counts and percentages, whilst continuous variables will be presented using the mean, median, standard deviation (SD), minimum, maximum, inter quartile range (IQR) and number of patients with an observation.

All analyses and data manipulations will be carried out using SAS version 9.4 or later.

4. List of Analyses

In this pilot trial, analyses are descriptive only, and there will be no formal statistical significance tests.

The outcomes of the pilot trial follow the SEAR (screened, eligible, approached, and randomised) framework for recording the recruitment process and reasons for non-participation[3]: Screening, to identify potentially eligible trial participants; Eligibility, assessed against the trial protocol inclusion/exclusion criteria; Approach, the provision of oral and written information and invitation to participate in the trial; and Randomised.

4.1 Outcomes

Descriptive statistics of the following outcomes will be reported for the entire pilot trial population:

1. The number of active sites, and the number of sites who have randomised participants

2. Implementation of trial procedures correctly as assessed by the number and type of protocol deviations recorded. The numbers of deviations will be tabulated, and deviations will be listed.

Loan JJM, *et al. BMJ Open* 2023; 13:e075187. doi: 10.1136/bmjopen-2023-075187

3. The numbers and proportions of patients (overall, and by site) who are screened, eligible, approached, uncertain, consented and randomised, which will be defined as:
   a) Screened: Number of patients screened with sufficient information to determine eligibility.
   b) Eligible: Screened patients meeting the trial's eligibility criteria (quantify any patients for whom this is uncertain separately). Proportion of screened patients who were eligible = b/a.
   c) Approached: Eligible patients who were approached for discussion (quantify any patients who were not approached and why or where this is unknown, separately). Proportion of eligible patients who were approached = c/b.
   d) Uncertain: Eligible patients who were approached about treatment with vs. without surgery and both doctor and patient were uncertain and therefore confirmed fully eligible (quantify any patients where only doctor, only patient, or neither is uncertain, or where this is unknown). Proportion of approached patients who are fully eligible = d/c.
   e) Consent: Fully eligible patients who have provided consent (quantify any not consented with reasons separately). Proportion of fully eligible patients who provide consent = e/d. Method of obtaining consent and who provided consent for the randomised study will be summarised.
   f) Randomised: Fully eligible patients consented, and randomised (quantify any patients who were not randomised and why or where this is unknown, separately). Proportion of eligible patients who were randomised = f/b.
   g) Withdrawn: Randomised patients who have withdrawn including who is withdrawing the participant, reason for and type of withdrawal (overall only, not by site).

   Proportions will be given with 95% confidence intervals (CI) (overall only, not by site).

4. Baseline characteristics will be summarised using descriptive statistics for eligible participants who were randomised versus eligible participants who were not randomised based on data collected at screening.
   - Source of screening
   - Speciality doing screening
   - Clinical history attributable to a brain cavernoma
     - Intracranial haemorrhage (ICH): one versus more than one vs none
     - Focal neurological deficit (FND): yes/no
     - Either ICH or FND
     - Epileptic seizure(s): yes versus no
   - Location of the symptomatic brain cavernoma (supratentorial lobar vs supratentorial deep grey matter vs brainstem vs cerebellum)
   - Time from most recent symptomatic event (months)

5. The number of participants randomised will be presented numerically overall and by site, and graphically overall over time.

6. The overall recruitment rate per month with 95% CI and the recruitment rate per site per month.

Descriptive statistics of the following baseline data and outcomes will be reported for (1) randomised participants overall and by randomised group, and (2) randomised participants by randomised group and stratification variable (preferred type of surgical management: neurosurgery versus stereotactic radiosurgery):

7. Baseline characteristics
    - Age
    - Gender
    - Ethnicity
    - Symptomatic brain cavernoma presentation
        - Brain cavernoma-related symptomatic ICH
        - Brain cavernoma-related symptomatic persistent or progressive FND
        - Brain cavernoma-related symptomatic epileptic seizure(s)
    - Symptomatic brain cavernoma details as reported by the investigator
        - Number of cavernomas (brain or spinal) (single versus multiple and median number in those with multiple)
        - Side of symptomatic brain cavernoma that could be managed surgically
        - Location of symptomatic brain cavernoma that could be managed surgically
        - Proximity of symptomatic brain cavernoma to surface of this location
        - Prior treatment of symptomatic brain cavernoma
    - Brain cavernoma certainty and imaging characteristics as reported by the study neuroradiologist
        - Received brain imaging required to confirm symptomatic brain cavernoma diagnosis and mode of presentation
        - Certainty about diagnosis of the symptomatic cavernoma
    - Intended type of surgical management agreed
    - Other medical history
    - Current medication
    - Current therapies
    - Modified Rankin scale score (adults only)
    - NIH stroke scale score total
    - Karnofsky Performance scale (adults only)
    - Lansky play performance scale (children only)
    - EQ-5D (Index and visual analogue scale (VAS))
    - Liverpool seizure severity scale (only patients with epileptic seizures in the preceding 4 weeks)

8. Intervention characteristics
    - Surgical management in participants undergoing neurosurgical excision
        - Type of anaesthesia
        - Craniotomy performed but cavernoma not found
        - Was neuro-navigation used
        - Was neurophysiological monitoring/stimulation used
        - Was intra-operative MRI performed
        - Was functional MRI performed

  - Grade of most senior neurosurgeon performing the procedure
  - Did the participant return to theatre for re-operation
  - Was post-operative MRI performed during this admission
- Surgical management in participants undergoing stereotactic radiosurgery
  - Location of stereotactic radiosurgery
  - Treatment prescription dose
  - Prescription isodose
  - Maximum dose
  - Paddick Conformity Index
  - Dose Gradient Index
  - Coverage
  - Treatment volume
  - Frame or mask-based
- Were any novel therapies used
  - Magnetic resonance thermography-guided laser interstitial thermal therapy used
  - Stereotactic laser ablation used
  - Other novel technique used
- Medical management
  - Physiotherapy
  - Speech and language therapy
  - Psychology
  - Occupational therapy

9. The number and proportion of randomised patients adherent to
   a) the allocated intervention based on
      i. intervention received and
      ii. whether the pre-specified type of surgical management (neurosurgery/radiosurgery) was the same as the type of intervention received
   b) follow-up based on completion of 6-month review CRF for those participants who are alive. Completeness of individual sections of the CRF will be summarised.

10. Completeness of data presented as the number and proportion with missing data for:
    a) Baseline. Defined as completion of the baseline CRF.
    b) Imaging. Defined as "Received brain imaging required to confirm symptomatic brain cavernoma diagnosis and mode of presentation" = yes, and at 6-months defined as "6-month MRI performed" = yes from the brain imaging data (not the CRF).
    c) Outcomes. Defined as completion of the follow-up review CRF for all follow-up time points (6, 12 and 18 months) that should have been reached by the participant. Completeness of individual sections of the CRF will be summarised.

11. Outcome event rates will be quantified using the number and proportion of participants with an event, the number of events, and the average event rate per participant per year. Outcome functional scores will be summarised descriptively and graphically to explore which has suitable statistical properties for use in a main phase trial (such as lack of floor/ceiling effects) for each time point available (6, 12 and 18 months). Clinical outcomes are:

    Primary

Loan JJM, *et al. BMJ Open* 2023; 13:e075187. doi: 10.1136/bmjopen-2023-075187

- Intracranial haemorrhage or new persistent/progressive focal neurological deficit due to brain cavernoma or surgical management (neurosurgery or stereotactic radiosurgery), whether fatal (leading to death within 30 days of the outcome event) or non-fatal.

Secondary

- Death not due to a primary clinical outcome
- Liverpool Seizure Severity Scale plus epileptic seizure frequency (number of seizures in the preceding four weeks, and attainment of one-year seizure freedom)
- Modified Rankin Scale (mRS) score
- National Institute of Health Stroke Scale Score (adult or paediatric)
- EQ-5D-5L in adults and EQ-5D-Y in children
- Karnofsky Performance Status scale in adults and Lanksy Play-Performance Scale in children

12. Follow-up imaging

6 month follow-up MRI

- MRI acquired as required by the protocol
- Is the symptomatic cavernoma that led to the participant's enrolment still present?
- Evidence of neurosurgical excision of the symptomatic brain cavernoma
  - If yes, was excision complete
- Evidence of stereotactic radiosurgery for the symptomatic brain cavernoma
  - If yes, change in cavernoma size, new signal change in surrounding brain, probable radio necrosis

Outcome Imaging

- Evidence of acute haemorrhage and locations

Outcomes 9-13 as listed in the protocol are not within the scope of this analysis plan and will be handled separately using data provided in this report to inform decisions for the design of the definitive main phase trial.

The analyses of the QuinteT recruitment intervention and health economics are also not within the scope of this analysis plan and will be handled separately.

4.2 Serious adverse events

Serious adverse events (SAEs) are reported if they are not outcome events or expected complications related to medical and surgical management.

SAEs will be summarised by treatment received and a listing will be produced detailing each event, and what happened to the patient subsequently.

5. Validation and QC

The statistical report will be read and sense-checked by a second statistician.

6. Data sharing

A file, or set of files, containing the final data will be prepared, along with a data dictionary. These will be made available to the Chief Investigator at the end of the analysis phase.

Following publication of the primary paper, a de-identified individual participant data set will be prepared for sharing purposes.

7. References

1. Eldridge SM, Chan CL, Campbell MJ, et al. CONSORT 2010 statement: extension to randomised pilot and feasibility trials. *BMJ* 2016; **355**: i5239.
2. Moultrie F, Horne MA, Josephson CB, et al. Outcome after surgical or conservative management of cerebral cavernous malformations. *Neurology* 2014; **83**(7): 582-9.
3. Wilson C, Rooshenas L, Paramasivan S, et al. Development of a framework to improve the process of recruitment to randomised controlled trials (RCTs): the SEAR (Screened, Eligible, Approached, Randomised) framework. *Trials* 2018; **19**(1): 50.
