# Supplementary material for: Feasibility of comparing medical management and surgery (with neurosurgery or stereotactic radiosurgery) with medical management alone in people with symptomatic brain cavernoma – protocol for the Cavernomas: A Randomised Effectiveness (CARE) pilot trial
Source: BMJ Open. 2023 Aug 9;13(8):e075187. doi: 10.1136/bmjopen-2023-075187 (PMC10414059; doi:10.1136/bmjopen-2023-075187)
Supplement: Supplementary data [file bmjopen-2023-075187supp005.pdf]

Version No 1.0

Effective Date 7 February 2023

CARE

Cavernomas A Randomised Effectiveness (CARE) pilot trial, to address the effectiveness of active treatment (with neurosurgery or stereotactic radiosurgery) versus conservative management in people with symptomatic brain cavernoma

Health Economic Analysis Plan (HEAP)

Version No	1.0
Date Finalised	7 February 2023
Author(s)	Alistair Bullen, Andrew Stoddart, Aileen Neilson
CI Name	Prof Rustam Al-Shahi Salman
CI Email address	Rustam.Al-Shahi@ed.ac.uk

Signatures	
Trial Lead Health Economist: Peter Hall	Date: 7 February 2023
Chief Investigator: Rustam Al-Shahi Salman	Date: 7 February 2023

Document Control		
Version No	Date	Summary of Revisions
1.0	22.11.2022	Initial Creation by author Alistair Bullen

Loan JJM, et al. BMJ Open 2023; 13:e075187. doi: 10.1136/bmjopen-2023-075187

Version No 1.0

Effective Date 7 February 2023

Supporting Internal Documents	Version No
CARE Protocol	2.0
CARE Statistical Analysis Plan	1.0

Version No 1.0

Effective Date 7 February 2023

Table of Contents

List of Abbreviations 4
1. Introduction 5
2. Objectives and Overview of Economic Evaluation 5
2.1. Overview of the Economic Evaluation 5
2.2. Primary Health Economic Objectives 6
2.3. Secondary Health Economic Objectives 6
3. Economic Principles 7
3.1. Cost Perspective 7
3.2. Time Horizon 7
3.3. Discount Rates 7
4. Data Collection & Processing 7
4.1. Analysis Software 7
4.2. Summary of Data Collection & Follow up Timing 7
4.2.1. Intervention 8
4.3. Resource Use and Cost Calculations 8
4.3.1. Base Year and Unit Cost Selection 8
4.3.2. Cost Calculations 10
4.4. Health Outcomes 10
4.4.1. QALY Outcome Calculation 10
5. Within Trial Analyses & Reporting 10
5.1. Scope of Analyses 10
5.2. Reporting Standards 11
5.3. List of Analyses 11
5.4. Assessment of Data Quality 12
6. Modelling 12
6.1. Existing Model 13
6.2. Assessment of Model Parameters for use in Current and Future Modelling 13
6.3. Dry Run Analysis 14
6.3.1. Outcomes 14
6.4. Results 15
7. References 15

Version No 1.0

Effective Date 7 February 2023

List of Abbreviations

Abbreviation	Full Name
CCM	Cerebral Cavernous Malformations
CEAC	Cost Effectiveness Acceptability Curve
CHEERS	Consolidated Health Economic Evaluation Reporting Standards
CT	Computerized Tomography
ECTU	Edinburgh Clinical Trials Unit
EQ-5D-5L	Euroqol Quality of Life Survey [5 Dimension, adult version]
EQ-5D-Y	Euroqol Quality of Life Survey [3 Dimension, child version]
FND	Focal Neurological Deficit
GP	General Practitioner
HCRU	Healthcare Resource Utilisation
HEAP	Health Economic Analysis Plan
ICER	Incremental Cost Effectiveness Ratio
ICH	Intracerebral Haemorrhage
MRI	Magnetic Resonance Imaging
NHS	[The UK] National Health Service
NICE	[The] National Institute for [Health and] Care Excellence
ONS	Office of National Statistics
PSA	Probabilistic Sensitivity Analysis
POD	Post-Operative Day
QALY	Quality Adjusted Life Year
SAP	Statistical Analysis Plan
UK	United Kingdom

Loan JJM, *et al. BMJ Open* 2023; 13:e075187. doi: 10.1136/bmjopen-2023-075187

Version No	1.0
Effective Date	7 February 2023

1. Introduction

This document details the criteria to be used for the definition of the analysis populations and the health economic methods for analysis of CARE (Trial Registration: ISRCTN Number: 41647111); Trial Funding: National Institute for Health and Care Research (NIHR) Health Technology Assessment (project no. 128694), a two-arm, parallel group randomised feasibility trial which aims to estimate the feasibility of performing a definitive main phase randomised controlled trial (RCT) comparing medical management to medical and surgical management (with neurosurgery or Gamma Knife stereotactic radiosurgery, according to their availability in clinical practice) for improving outcomes for people with symptomatic brain cavernoma.

The aim is to randomise approximately 60 participants (from sites in the UK and Ireland) to groups in a 1:1 ratio, to medical management alone, or medical and surgical management, stratified by preferred type of surgical management. If there is no clear preference for the type of surgical management, and both are available, the patient will be randomly allocated to either neurosurgery or stereotactic radiosurgery, and then randomised between medical management alone, or medical and surgical management (detailed in section 3.1 of the trial protocol).
The pilot phase of CARE will be submitted for publication and reported according to the CONSORT 2010 extension to randomised pilot and feasibility trials.

The strategy set out here to guide the CARE health economic analyses, is intended to establish the rules that will be followed as closely as possible, when analysing and reporting the CARE trial health economic analyses. The principles set out here follow current published best practice for trial based economic assessments and recommended guidance regarding the content of the HEAPs for clinical trials.[1] This HEAP document has been written based on information contained in the trial protocol version 2.0, dated 22nd March 2021, and Statistical Analysis Plan (SAP) version 1.0, 12/12/2022. The HEAP is designed to ensure that there is no conflict with the protocol and associated statistical analysis plan and it should be read in conjunction with them.

Any deviations from the health economic analysis plan (described in this document) will be detailed and justified fully in the final report of the trial.

2. Objectives and Overview of Economic Evaluation

2.1. Overview of the Economic Evaluation

We aim to pilot the data collection methods for the CARE trial, and their assess suitability for use in a future full-scale trial providing descriptive statistics only, and an assessment of the completeness of surveys.

If suitable, we aim to adapt an existing decision analytic Markov health economic model by Rinkel et al, which presently only models QALYs, to further include costs enabling full economic evaluation to be conducted.[2] We plan to assess the appropriateness of each parameter in the model, augmenting with trial data as necessary and where possible, making recommendations for future use or development in a full-scale trial. If deemed viable, we will then undertake a dry run of the updated model using the updated parameters by way of proof of concept, to provide highly provisional cost-utility estimates based on NICE reference case recommendations and estimate plausible ranges of incremental costs and QALYs and understand the main driver parameters within the model.[3]

The broader aim is to support the case for a full scale RCT in the setting that has the potential to identify the most cost-effective solution for clinical practice that can improve resource allocation efficiency in order to maximise the benefits provided by the NHS.

2.2. Primary Health Economic Objectives

The primary health economic objectives as defined in the CARE protocol are:

1. Design and test optimal methods for capture of resource use and cost data in community NHS settings, NHS secondary care, participants' out of pocket expenses and carer costs.
2. Estimate expected effect size and variance of relevant outcomes including health-related quality of life (utility) and quality-adjusted life years (QALYs)
3. Identify and measure the potential cost implications of surgical management of cavernomas.

These relate to and comprise of the within-trial analysis component of the study, which focuses on assessment of the quality of data collected during the observed follow-up period of the trial.

2.3. Secondary Health Economic Objectives

The secondary objective of the health economics analysis are:

4. To test the effect of updated parameters informed by the results of the primary health economic analysis on a previously published decision analytical model in the same setting.[2]
5. Provide recommendations for revisions to the model to aid future definitive trial design.

These relate to and comprise of the modelled analysis component of the study, which focuses on assessment of the feasibility of simulating longer term outcomes, beyond those of the observable trial period.

Version No	1.0
Effective Date	7 February 2023

3. Economic Principles

3.1. Cost Perspective

The primary perspective for analysis is the healthcare payer (NHS) perspective. Secondary analyses include wider societal perspective which includes some personal costs borne by patients as well as community care costs.

3.2. Time Horizon

Time horizon for within-trial elements of the analysis will be 18 months, reflecting the observed time frame from baseline to last follow-up. Time horizon for economic modelling will be 5 years, to include the simulated extrapolation beyond the observed trial time horizon, match the time period used by the original model, and to facilitate meaningful comparisons between original and adapted (CARE) models.

3.3. Discount Rates

Base-case discount rates will be set to 3.5% for both costs and outcomes, following the NICE reference case recommendations.[3]

4. Data Collection & Processing

4.1. Analysis Software

The primary within trial analyses (Objectives 1 to 3) will be performed on STATA 17.[4] Secondary analysis re-purposing an existing decision analytical model (Objectives 4 and 5) is expect to be completed on R Studio.[5] Additional analysis may also be completed on Microsoft Excel and TreeAge.[6,7]

4.2. Summary of Data Collection & Follow up Timing

Table 1 presents data collection for items and corresponding time points relating specifically to the within trial health economics analysis. Patient utility values will be collected using the EQ-5D-5L measure for adults[8] and EQ-5D-Y[9,10] measure in children. Healthcare resource use and socioeconomic data will also be collected from information gathered in the form of participant self-reported questionnaires.

Loan JJM, et al. BMJ Open 2023; 13:e075187. doi: 10.1136/bmjopen-2023-075187

Version No 1.0

Effective Date 7 February 2023

Table 1: Summary of Health Economic Data Collection based on baseline and follow-up

Item	Time since baseline			
	Baseline	6-month	12 -month	18 month
**Health Utility data**				
EQ-5D-5L (adults only)	✓	✓	✓	✓
EQ-5D-Y (children only)	✓	✓	✓	✓
**Socioeconomic data***				
Employment data	✓	✓	✓	✓
Education data	✓	✓	✓	✓
Informal Care data	✓	✓	✓	✓
**Healthcare Resource Use**				
In-patient stays		✓	✓	✓
Out-patient service use		✓	✓	✓
Hospital tests		✓	✓	✓
Community and primary care		✓	✓	✓

* number of days lost due ill health, days of care provided by family and friends

4.2.1. Intervention

A case report form (CRF) is completed after the intervention, with data collected depending on the intervention performed (neurosurgical excision or stereotactic radiosurgery). Date of hospital admission and discharge for surgical management are collected for both interventions, and for patients who receive neurosurgical excision the type of ward attended (e.g. Adult, Paediatric, Neurology/Neurosurgery ,Other) is recorded. This information will be used to guide the selection of appropriate unit costs (from standard UK published literature sources) to assign to each type of surgical management intervention. We will also consult with relevant NHS service business managers as an alternative information source to estimate the costs associated with the different surgical treatment options.

4.3. Resource Use and Cost Calculations

4.3.1. Base Year and Unit Cost Selection

Base year for all costs will be selected as the latest financial year for which price weight reports are available at time of analysis and at least one patient provided data. A unit cost (in GBP) for each item for this base year will be sourced prior to analysis. As additional unit cost sources may be published

Loan JJM, *et al. BMJ Open* 2023; 13:e075187. doi: 10.1136/bmjopen-2023-075187

by time of analysis, unit costs will be identified close to time of analysis, prior to unblinding, and detailed in an updated HEAP signed off by PH & RASS. Table 2 below details the variables recorded in the relevant CRF, associated cost category, and anticipated sources for unit costs to be prioritised for each item. Alternatives unit costs maybe sourced for those unavailable or not deemed generalisable to the trial population/context at time of analysis.

Table 2: Summary of costs and expected correspond sources.

Item	Units	Anticipated Source*
**Direct Intervention Related Costs (In-Patient Hospitalisation)**		
Neurosurgical excision		NHS Reference costs[11]
Stereotactic excision		NHS Reference costs[11]
Adult ward in-patient stay (Post neurosurgical excision)	Per night	NHS Reference costs[11]
Paediatric ward in-patient stay (Post neurosurgical excision)	Per night	NHS Reference costs[11]
Neurology/Neurosurgery ward in-patient stay (Post neurosurgical excision)	Per night	NHS Reference costs[11]
Other ward in-patient stay (Post neurosurgical excision)	Per night	NHS Reference costs[11]
**In-patient Hospital Services**		
Hospital in-patient stay	Per night	NHS Reference costs[11]
Other unscheduled hospital or A&E attendance	Per attendance	NHS Reference costs[11]
**Out-patient Hospital Service**		NHS Reference costs[11]
Neurologist service	Per clinic/phone consultation	NHS Reference costs[11]
Surgeon service	Per clinic/phone consultation	NHS Reference costs[11]
Specialist nurse service	Per clinic/phone consultation	NHS Reference costs[11]
**Hospital Tests**		NHS Reference costs[11]
MRI Scan	Per clinic/phone consultation	NHS Reference costs[11]
CT Scan	Per clinic/phone consultation	NHS Reference costs[11]
**Community and Primary Care Services**		
GP surgery (doctor)	Per clinic/phone consultation	PSSRU[12]
GP surgery (nurse)	Per clinic/phone/home consultation	PSSRU[12]
NHS 24/111	Per clinic/phone/home consultation	Pope et al. [13]
Out of hours GP	Per clinic/phone/home consultation	PSSRU[12]
District nurse	Per clinic/phone/home consultation	PSSRU[12]
Nurse (other)	Per clinic/phone/home consultation	PSSRU[12]
Psychologist	Per clinic/phone/home consultation	PSSRU[12]
Physiotherapist	Per clinic/phone/home consultation	PSSRU[12]
Dietician	Per clinic/phone/home consultation	PSSRU[12]
Occupational therapist	Per clinic/phone/home consultation	PSSRU[12]
**Employment and Support (Indirect Costs)**		
Productivity losses (patient time off work due to health problems)	Per day	National average wage according to ONS[14]

Productivity losses (informal carers time off work to support/help patient)	Per day	National average wage according to ONS[14]

* Where a given item has multiple consultation types (e.g. clinic/phone/home), separate unit costs will be identified for each.

4.3.2. Cost Calculations

Each item of resource use will be multiplied by its unit cost to estimate a cost per patient, plus a total cost over all follow-up time points. This will be undertaken separately for each trial arm.

The following total cost categories will be calculated:

1. Mean per patient NHS costs will be calculated as the sum of mean cost per patient pertaining to direct intervention, in-patient hospital services, out-patient hospital service, hospital tests, and utilisation of community and primary care services.
2. Mean per patient wider societal costs will be calculated as the sum of mean cost per patient pertaining to NHS costs (as per 1.) plus lost income from days taken off of work by patients and informal carers.

4.4. Health Outcomes

4.4.1. QALY Outcome Calculation

Following NICE guidance, health utilities will be calculated for each patient based on their EQ-5D-5L or EQ-5D-Y at each time point if they were issued, and derived using the recommended UK EQ-5D-5L to 3L "Crosswalking" algorithm,[15] or based on sensitivity analysis between possible alternative scoring algorithms for the UK EQ-5D-Y.

QALYs will be calculated from these health utility values using an area-under-the-curve technique.[16]

5. Within Trial Analyses & Reporting

5.1. Scope of Analyses

We only aim to assess the suitability of the data collected for use in a future trial, and/or economic model. As such, calculations of incremental cost-effectiveness Ratios (ICERs) will not be undertaken on the within trial proportion of the analysis. Some preliminary calculations may however be undertaken as part of the modelling proportion of the sub study, see section 6. The main outputs from the within trial analysis will instead be the expected effect size and variance of relevant outcomes including health related quality of utility, QALYs, and cost factors.

All analyses will be based on the intention to treat (ITT) principle with patients analysed according to allocated treatment, irrespective of whether they adhered to the allocated treatment, in the group to which they were allocated.

5.2. Reporting Standards

Results will be presented in accordance to guidance set out in the Consolidated Health Economic Evaluation Reporting Standards (CHEERS).[17]

5.3. List of Analyses

As CARE is a pilot trial, only descriptive statistics will be provided with no formal statistical significance tests.

Completeness of the following outcomes will be summarised for each time point (6, 12 and 18 months) and each trial arm, with completion defined as the number and percentage of responses from participants that should have been reached at that time point.

i. Each resource use item listed in Table 2 (see section 4.3.1)

ii. Each EQ-5D-5L or EQ-5D-Y sub-scale (Mobility, Self-Care, Usual-Activities, Pain and Discomfort, and Anxiety and Depression).

The following outcomes will be reported for each trial arm:

iii. Mean rates of utilisation per patient, and associated standard deviation of each resource use item listed in Table 2 (see section 4.3.1), at each time point (6, 12 and 18 months) and total over all time points.

iv. Mean cost (calculated as per section 4.3.2) of each resource use item listed in Table 2 (see section 4.3.1) per patient and associated standard deviation totalled over all time points.*

v. Mean total costs (calculated as per section 4.3.2) per patient for each category of cost.*

vi. Mean utility scores (calculated as per section 4.4.1) per patient and associated standard deviation at each time point (6, 12 and 18 months).

vii. Mean QALYs per patient (calculated as per section 4.4.1) and associated standard deviation.*

* Cost and QALY figures (Outcomes iv., v., and vii.) may be calculated accounting for missing data e.g. through imputation, with the selection of a specific method being informed by the quantity and pattern of missingness present and , subject to data quality assessment (see Section 5.4).

Subject to data quality (See Section 5.4), regression analyses adjusting for baseline may be explored for total costs and QALYs (Outcomes v. and vii.).

Subgroup analysis (for items i-vii above) considering age-group (adults vs children) and by intervention type (neurosurgery or stereotactic radiosurgery) will also be conducted subject to adequate numbers

being available. Finally, costs related to the specific health states defined in the previously developed QALYs (only) model by Rinkel et al (see section 6.1 below) will be reported if identifiable from the pilot data collected.

5.4. Assessment of Data Quality

A qualitative assessment of missingness and data quality pertaining to the health economic analysis, from outcomes i. and ii. In Section 5.3., will be produced by the health economics team. Analysts will provide an expert assessment of the data quality with respect to:

- Suitability for use in future definitive trials in light of larger sample sizes.
- Adaptation for use in parameters of the economic modelling in Section 6, and any similar modelling alongside a hypothetical definitive future trial in light of larger sample sizes.

We will also make recommendations around appropriate forms of imputation that may be necessary in future trials. QALY and total cost calculations are composite variables by their nature. As such even single missing items on any resource or utility observation at any time point can render a participants QALY or total cost figures incalculable, without some form of imputation. Assessment of data quality will include consideration of what form of imputation may be necessary in a future main phase definitive trial. However as the regressions needed for more advanced imputation techniques would be underpowered, at most, simple mean imputation may be applied at the analysts discretion.

6. Modelling

Subject to data quality assessment (see Sections 5.4, and 6.2), an existing model by Rinkel et al[2] will be rebuilt, and adapted to incorporate trial data. The latter being important in order to add cost elements in particular, as the existing model simulates effectiveness in terms of QALYs only.

The purpose of the model will be to:

1. Create a model structure for potential adaptation and reuse alongside future definitive trial.
2. Undertake a proof of concept dry run analysis to identify any issues in the model and make recommendations for adaptation for use in any future definitive trial.
3. If data quality are suitable, provide highly provisional early estimates of cost-utility of medical management alone vs medical and surgical management (with neurosurgery or Gamma Knife stereotactic radiosurgery, according to their availability in clinical practice) for the treatment of symptomatic brain cavernoma.

To maximise UK policy relevance, this adaptation will follow NICE reference case recommendations[3] where possible including: Adoption of an NHS and PSS (personal social service) costing perspective for primary analyses; cost-utility approach (results presented in terms of incremental cost per QALY derived from EQ-5D-5L); discount rate of 3.5% for both costs and QALYs; and the use of probabilistic sensitivity analysis (PSA), to generate cost effectiveness acceptability curves (CEACs).[18] Any exceptions to reference case methodology will be noted and justified. Time horizon for analysis will be 5 Years (see Section 3.2).

6.1. Existing Model

A model schematic, including diagrams, parameter estimates and sources, and modelling assumptions can be found in the technical appendix to Rinkel et al.[2] By way of overview, the model compares three treatment arms (Conservative Management, Stereotactic radiosurgery, and neurosurgical excision) using a 5 year Markov model, with 3 primary health states (Well, Disabled and Death). Well and Disabled health states are subdivided into proportions with about without seizures and/or ICH. The model simulated three cohorts: (patients with brainstem cerebral cavernous malformations(CCM), patients with non-brainstem CCM presenting with intracerebral haemorrhage (ICH)/ focal neurological deficit FND, and patients presenting with epilepsy. Model parameters are populated using systematic review of published studies of CCM from the inception of Medline and Embase to December 2016. Primary outcomes from the model are expected number of QALYs, and ICH recurrence risk.

6.2. Assessment of Model Parameters for use in Current and Future Modelling

A table of model parameters will be generated detailing:

a. The parameter name and description.

b. Desired statistical distribution for the parameter for use in a Method of Moments approach to enable PSA.[19]

c. Candidate values and sources (trial data, or existing model) where available. Where multiple sources are identified, each will be listed.

d. A qualitative expert assessment of the suitability of the available source(s), accounting for generalisability to patient population and context, and a statement of which parameter is preferred (where a choice exists), for (i) current modelling utilising pilot data, and (ii) future modelling utilising data from a hypothetical future definitive scale trial. Note that it is possible that recommendations for current modelling source prioritisation may differ due to expected larger sample sizes in a future trial.

Results for d. may be reported as body text if the discussion is too large to be included in the table.

A qualitative expert assessment in the form of a short interim report of the model structure as a whole will then be undertaken highlighting any areas of weakness, with a focus on parameters which may not be suitable from either source (existing model or trial data) and with recommendations for future literature reviews which may be needed to populate them if necessary. Such reviews may be undertaken, subject to available time, at the analysts discretion.

6.3. Dry Run Analysis

Subject to suitability of available parameters, the model[2] will be rebuilt in R and RStudio[5] with the addition of cost parameters linked to key health states and transitions. The model will be parameterised applying the recommendations for best current available data from the interim report generated by process described in Section 6.2.

Any adaptation to the model structure from that of the original which arise as necessary during the models development will be noted and justified, with a new model schematic diagram generated.

6.3.1. Outcomes

Outcomes for the model will be:

A. Mean QALYs per patient for each trial arm, and difference in mean QALYs per patient between trial arms (intervention minus control). Note that the method for calculating QALYs will depend on data available (see Section 6.2, though preference will be given to calculation via NICE recommended[3] EQ-5D utilities where available)

B. Mean NHS cost per patient for each trial arm, and difference in mean NHS cost per patient between trial arms (intervention minus control).

C. ICER(s) in terms of incremental cost per QALY (intervention vs control, calculated as [A]/[B] above).[16,19]

D. A CEAC, generated via PSA utilising a method of moments approach[19], with point estimates of likelihood of each arm being the most cost-efficient at NICE recommended thresholds of £20k, and £30k per QALY.

Note we will not undertake value of information analysis (VoI) as this assumes all data to be generalisable to the patient population and context, and we do not anticipate this to be the case. However, we will conduct a limited range of deterministic and probabilistic (one-way) sensitivity analysis in order to help understand the influence and implications of important model input parameters.

6.4. Results

Outcomes A – D in section 6.3.1 will be reported, however these are expected to carry strong caveats that they are provisional results only.

A short report summarising the findings from Section 6.3.1 and experiences developing and running the model will be created by the analyst, with support from senior health economists, which will provide recommendations for developments for the model for use alongside any future definitive trial such as:

- Changes to model structure.
- Alternative data sources for parameterisation (Including need for literature reviews(s)).
- Any concerns about the model, or matters arising in its development so far.

7. References

1. Thorn JC, Davies CF, Brookes ST, Noble SM, Dritsaki M, Gray E, et al. Content of Health Economics Analysis Plans (HEAPs) for trial-based economic evaluations: expert Delphi consensus survey. Value Health. 2021;24(4):539– 47.
2. Rinkel LA, Salman RAS, Rinkel GJ, and Greving JP. Radiosurgical, neurosurgical, or no intervention for cerebral cavernous malformations: a decision analysis. International Journal of Stroke. 2019; 14(9), pp.939-945.
3. National Institute of Health and Care Excellence. NICE Health Technology Evaluations: The Manual. NICE 2022.
4. StataCorp. 2021. Stata Statistical Software: Release 17. College Station, TX: StataCorp LLC.
5. RStudio Team (2020). RStudio: Integrated Development for R. RStudio, PBC, Boston, MA URL http://www.rstudio.com/.
6. Microsoft Corporation. Microsoft Excel [Internet]. 2018. Available from: https://office.microsoft.com/excel
7. TreeAge Pro 2021, R1. TreeAge Software, Williamstown, MA; software available at http://www.treeage.com.
8. Herdman M, Gudex C, Lloyd A, Janssen M, Kind P, Parkin D, et al. Development and preliminary testing of the new five-level version of EQ-5D (EQ-5D-5L). *Qual Life Res.* 2011, Vol. 20, 10, pp. 1727-1736.
9. Wille N, Badia X, Bonsel G, Burström K, Cavrini G, Devlin N et al. Development of the EQ-5D-Y: a child-friendly version of the EQ-5D. Qual Life Res 2010;19:875–886. 10.
10. Ravens-Sieberer U, Wille N, Badia X, Bonsel G, Burström K, Cavrini G,et al. Feasibility, reliability, and validity of the EQ-5D-Y: results from a multinational study. Qual Life Res 2010;19:887–897.
11. Department of Health, The. Reference Costs Publications. The Department of Health 2021. https://www.gov.uk/government/collections/nhs-reference-costs

12. Curtis L. Unit Costs of Health & Social Care 2021.Kent: Personal Social Services Research Unit 2021.
13. Pope C, Turnbull T, Jones J, Prichard J, Rowsell A, and Halford S. Has the NHS 111 urgent care telephone service been a success? Case study and secondary data analysis in England. BMJ Open 2017; 7:e014815. doi: 10.1136/ bmjopen-2016-014815.
14. Office of National Statistics. https://www.ons.gov.uk/
15. Van Hout B, Janssen M, Feng Y et al. Interim scoring for the EQ 5D 5L: Mapping the EQ 5D 5L to EQ 5D 3L value sets. Value in Health. 2012; 15: 708-15.
16. Drummond MF, Sculpher MJ, Claxton K, Stoddart GL, and Torrance GW. Methods for the Economic Evaluation of Health Care Programmes. Oxford: Oxford University Press, 2015.
17. Husereau D, Drummond M, Petrou S, Carswell C, Moher D, Greenberg D, et al. Consolidated health economic evaluation reporting standards (CHEERS)-explanation and elaboration: a report of the ISPOR health economic evaluation publication guidelines good reporting practices task force. Value Health. 2013;16:231–50.
18. Glick HA, Doshi DA, Sonnad SS, and Polsky D. Economic Evaluation in Clinical Trials. Oxford: Oxford University Press, Incorporated, 2014.
19. Briggs A, Sculpher M, and Claxton K. Decision Modelling for Health Economic Evaluation. Oxford: Oxford University Press, Incorporated, 2006.
