# Supplementary material for: Feasibility of comparing medical management and surgery (with neurosurgery or stereotactic radiosurgery) with medical management alone in people with symptomatic brain cavernoma – protocol for the Cavernomas: A Randomised Effectiveness (CARE) pilot trial
Source: BMJ Open. 2023 Aug 9;13(8):e075187. doi: 10.1136/bmjopen-2023-075187 (PMC10414059; doi:10.1136/bmjopen-2023-075187)
Supplement: Supplementary data [file bmjopen-2023-075187supp006.pdf]

# CARE Trial Data Monitoring Committee Charter

| | |
|---|---|
| **Study Title:** | Cavernomas A Randomised Effectiveness (CARE) pilot trial, to address the effectiveness of active treatment (with neurosurgery or stereotactic radiosurgery) versus conservative management in people with symptomatic brain cavernoma |
| **Funder and funder reference:** | National Institute for Health Research Health Technology Assessment Programme - NIHR128694 |
| **Chief Investigator:** | Prof Rustam Al-Shahi Salman |
| **Co-Sponsors:** | University of Edinburgh & NHS Lothian |
| **Sponsor reference:** | AC20171 |
| **Trial Registration Reference(s):** | ISRCTN41647111 |
| **REC reference:** | 21/YH/0046 |
| **Charter Version Number and Date:** | V2.0 (24Jan2023)<br>*Based on sponsor template CR015-T01 v3.0* |



Loan JJM, *et al. BMJ Open* 2023; 13:e075187. doi: 10.1136/bmjopen-2023-075187

## Approval Signatures:

The following individuals, by providing their signatures, indicate their understanding of and willingness to comply with the roles and responsibilities assigned to them in this Charter.

1. DMC Chair:

| | | |
|---|---|---|
| ______________ | ______________ | _ _ / _ _ /_ _ _ _ |
| JOHN BAMFORD | SIGNATURE | DATE |

2. DMC Member:

| | | |
|---|---|---|
| ______________ | ______________ | _ _ / _ _ /_ _ _ _ |
| DAVID MENDELOW | SIGNATURE | DATE |

3. DMC Member:

| | | |
|---|---|---|
| ______________ | ______________ | _ _ / _ _ /_ _ _ _ |
| NIGEL BAKER | SIGNATURE | DATE |

4. Chief Investigator:

| | | |
|---|---|---|
| ______________ | ______________ | _ _ / _ _ /_ _ _ _ |
| RUSTAM AL-SHAHI SALMAN | SIGNATURE | DATE |

5. Trial Statistician:

| | | |
|---|---|---|
| ______________ | ______________ | _ _ / _ _ /_ _ _ _ |
| PRINT NAME | SIGNATURE | DATE |



Loan JJM, *et al. BMJ Open* 2023; 13:e075187. doi: 10.1136/bmjopen-2023-075187

Table of Contents

1 Introduction ..... 4
2 Roles and Responsibilities ..... 4
3 Before or early in the trial ..... 5
4 Composition ..... 6
5 Relationships ..... 6
6 Organisation of DMC Meetings ..... 7
7 Trial Documentation and Procedures to Ensure Confidentiality and Proper Communication ..... 8
8 Decision Making ..... 9
9 Reporting ..... 10
10 After the Trial ..... 11

## 1 Introduction

This Charter is for the Data Monitoring Committee (DMC) for the Cavernomas A Randomised Effectiveness (CARE) pilot trial, a pilot randomised controlled trial (RCT) which aims to assess the feasibility of conducting a definitive main phase RCT to address the research question "How effective is active treatment (with neurosurgery or stereotactic radiosurgery) versus conservative management in people with symptomatic brain cavernoma?". The trial objectives are to:

1. Engage a collaboration of specialists and patient advocacy groups in the UK and Ireland.
2. Establish a pilot RCT, with an embedded qualitative study to understand the anticipated recruitment processes and address any barriers.

   Assess the feasibility of performing a definitive main phase of the RCT.

The Charter will define the primary responsibilities of the Data Monitoring Committee (DMC) for the CARE pilot trial, its membership, and the purpose and timing of its meetings. The Charter will also provide the procedures for ensuring confidentiality and proper communication, the statistical monitoring guidelines to be implemented by the DMC, and an outline of the content of the Open and Closed Reports that will be provided to the DMC.

The trial will be conducted in accordance with sponsor SOPs: (https://www.accord.scot/research-access/resources-researchers/sop).

The contents of the Charter are based on the NIHR Research Governance Guidelines for Data Monitoring Committees: (https://www.nihr.ac.uk/documents/research-governance-guidelines/12154).

## 2 Roles and Responsibilities

The DMC is an independent multidisciplinary group consisting of clinicians and statisticians that, collectively, have experience/expertise in the management of patients with the condition relevant to trial and in the conduct and monitoring of randomised clinical trials. University of Edinburgh insurance indemnifies DMC members for their work on the committee.

The specific roles of the DMC include:

- The DMC will be responsible for:
  - Safeguarding the interests of trial participants, potential participants, investigators and sponsor, ensuring that the safety, rights and well-being of the trial participants are paramount
  - Assessing the safety and efficacy of the interventions during the trial, with due allowance for this being a feasibility study
  - Reviewing external evidence with an impact on risk/benefit balance, with due allowance for this being a feasibility study



Loan JJM, et al. BMJ Open 2023; 13:e075187. doi: 10.1136/bmjopen-2023-075187

- Monitoring the overall conduct of the clinical trial

- The DMC will provide recommendations about stopping, modifying or continuing the trial to the Trial Steering Committee (TSC).

- The DMC will contribute to enhancing the integrity of the trial, and may also formulate recommendations relating to the selection, recruitment, or retention of participants, or their management, or to improving their adherence to protocol-specified regimens and retention of participants, and the procedures for data management and quality control.

- The DMC will consider the need for any interim analysis advising the TSC regarding the release of data and/or information

- On rare occasions when the DMC chair might be asked, through the chair of the TSC, by the Funder to provide advice based on a confidential interim or futility analysis if serious concerns are raised about the viability of the study or if the research team are requesting significant extensions, but this in unlikely in a feasibility setting.

- The DMC will be notified of all changes to the protocol or to study conduct. The DMC concurrence will be sought on all substantive recommendations or changes to the protocol or study conduct prior to their implementation.

## 3 Before or early in the trial

All potential DMC members will have sight of the protocol before the first DMC meeting. Before recruitment begins, the trial will have undergone review by the sponsor and a research ethics committee. Therefore, if a potential DMC member has major reservations about the trial (e.g. the protocol or the logistics) they should report these to the CI and may decide to decline the invitation to join. DMC members should be constructively critical of the ongoing trial, but also supportive of aims and methods of the trial.

The DMC will aim to meet before or close to the start of recruitment to the trial, to discuss the protocol, methods of providing information to and from the DMC, frequency and format of meetings, relationships with other committees and have the opportunity to clarify any aspects with the CI and Co-Chief Investigator. DMC input into the protocol will be discussed with the CI before deciding what protocol updates need to be implemented.

Members and observers of the DMC will not be asked to formally sign a contract but should formally register their assent by confirming (1) that they agree to be a member of the DMC and (2) that they agree with the contents of this Charter by signing and dating the required form (Appendix 1).

## 4 Composition

DMC members were selected and approved by the funder in accordance with NIHR Research Governance Guidelines (V1.0 February 2019).

The **members** of the DMC are listed below.

| Name of Member | Role in DMC | Responsibility |
|---|---|---|
| Dr John Bamford | Independent Chair | Provide independent neurological expertise |
| Prof David Mendelow | Independent member | Provide independent neurosurgical expertise |
| Mr Nigel Baker | Independent member | Provide independent statistical expertise |

In addition, the following individuals will also be involved in DMC meetings:

| Name | Trial Role | Responsibility |
|---|---|---|
| Prof Rustam Al-Shahi Salman | Chief Investigator | Inform DMC of any relevant updates |
| Mr Neil Kitchen | Co-chief investigator | Neurosurgical lead |
| Prof Steff Lewis | Statistician | Blinded trial statistician |
| Ms Jacquie Stephen | Statistician | Unblinded trial statistician |
| Dr Laura Forsyth | Trial Manager / Facilitator | Co-ordinate meetings and facilitate the group |

See section 7 for more information on the roles of the blinded and unblinded trial statisticians.

DMC membership is normally for the duration of the trial. If any member leaves the DMC during the course of the trial, the Sponsor, in consultation with the TSC and/or Investigators will promptly appoint their replacement.

## 5 Relationships

### DMC/ TSC relationship

The primary DMC reporting line is via the Chair to the TSC. The DMC will be advisory to the TSC. The TSC will be responsible for promptly reviewing the DMC recommendations, to decide whether to continue or terminate the trial, and to determine whether amendments to the protocol or changes in study conduct are required.

### Payments to DMC members



Loan JJM, *et al. BMJ Open* 2023; 13:e075187. doi: 10.1136/bmjopen-2023-075187

If required, standard travel and accommodation costs will be paid to members of the DMC. No other payments or rewards will be given.

**Competing Interests**

Any competing interests, either real or potential, should be disclosed before DMC meetings (see Appendix 1). These are not restricted to financial matters, involvement in other trials or intellectual investment could be relevant. Although members may well be able to act objectively despite such connections, complete disclosure enhances credibility.

## 6 Organisation of DMC Meetings

**Meeting Frequency**

Responsibility for calling and organising DMC meetings lies with the Chief Investigator, in association with the Chair of the DMC, who will be assisted by the Trial Manager/Facilitator. The DMC should meet at least annually, or more often as appropriate, and meetings should be timed so that reports can be fed into the TSC.

**Meeting Format and Attendance**

Sessions involving only DMC membership (but often including the unblinded statistician as well, as a non-voting member) called Closed Sessions will be held to allow discussion of confidential data from the clinical trial, including information about the relative efficacy and safety of interventions. In order to ensure that the DMC will be fully informed in its primary mission of safeguarding the interest of participating patients, the DMC will be unblinded in its assessment of safety and efficacy data. During these sessions, the DMC will develop a consensus on its list of recommendations, including that relating to whether the trial should continue. Attendance at DMC meetings by non-members is at the discretion of the Chair

DMC members and all other participants in the closed session of DMC meetings and the production of unblinded reports are expected to maintain confidentiality, and will refrain from revealing to the Trial Steering Committee, or any other party, information that would lead to compromising the integrity of the trial unless such release is required to protect patient safety.

In order to allow the DMC to have adequate access to information provided by the trial investigators, or by members of the regulatory authorities, a joint session between these individuals and DMC members (called an Open Session) will be held before the Closed Session. The trial Chief Investigator, Trial Statistician and Trial Manager will be available in-person or by phone for an open session at the beginning of the meeting, and will be available at the end of the meeting to answer any urgent questions. If necessary, a further Open Session can be held, on request either in the middle or end of the Closed Session. Open sessions give the DMC an opportunity to query these individuals about issues that have arisen during their review in the initial Closed Session. With this format, important interactions are facilitated through which problems affecting trial integrity can be identified and resolved.

Effort will be made to ensure that all members can attend. The CI must try to attend



Loan JJM, et al. BMJ Open 2023; 13:e075187. doi: 10.1136/bmjopen-2023-075187

all meetings, especially if major actions are expected. In the case of face to face meetings, members who cannot attend in person will be encouraged to participate by teleconference/videoconference. If DMC members cannot attend meetings by tele-/video-conference, they will be encouraged to send comments in advance via email.

Meetings will be held either in person, by video-conference (e.g. Zoom, MS Teams) or by teleconference. Major trial issues may need to be dealt with between meetings, by phone, video-conference or by email. DMC members should be prepared for such instances. There may be occasions when the Sponsor or the Funder will wish to organise and administer these meetings for particular projects. This is unlikely, but the NIHR reserves the right to attend any meeting therefore should be included in relevant invitations and also reserves the right to convene a meeting of the TSC in exceptional circumstances.

### Quoracy

The minimum quoracy for a meeting to conduct business is 67% (two thirds) of appointed members. If, at short notice, any DMC members cannot attend then the committee may still meet if at least 2 members including the Chair will be present. If the DMC is considering a major action after such a meeting the Chair should communicate with the absent members, including the CI, as soon after the meeting as possible to check they agree. If they do not, a further meeting should be arranged with the full DMC.

### Non-attendance

DMC members who will not be able to attend the meeting should pass comments to the committee Chair in advance for consideration during the discussion. If a member does not attend a meeting or provide comments when requested between meetings, it will be ensured that the member is available for the next meeting. If a member does not attend the next meeting or provide comments when next requested, they will be asked if they wish to remain part of the DMC. If an independent member does not attend a third meeting, strong consideration will be given to replacing this member.

## 7 Trial Documentation and Procedures to Ensure Confidentiality and Proper Communication

To enhance the integrity and credibility of the trial, procedures will be implemented to ensure the DMC has sole access to evolving information from the clinical trial regarding comparative results of efficacy and safety data, aggregated by treatment arm. An exception will be made to permit access to an unblinded statistician who will be responsible for creating the closed report and sending it to the DMC. The Chief Investigator will provide the chair of the DMC with information on any serious unexpected adverse reactions to the study drug, and will also be responsible for satisfying the standard requirements for reporting of relevant events to the regulatory authorities.

### Meeting Content and Reports



Loan JJM, *et al. BMJ Open* 2023; 13:e075187. doi: 10.1136/bmjopen-2023-075187

At the first DMC meeting, the committee will provide an advisory review of scientific and ethical issues relating to study design and conduct, discuss the functioning of the DMC and discuss the format and content of the Open and Closed Reports that will be used to present trial results at subsequent DMC meetings.

The following intended content may be included in the reports:

- *Intended content of material to be available in open sessions.*
  Open Reports, available to all who attend the DMC meeting, will include any major protocol changes, data on recruitment and baseline characteristics; pooled data on eligibility violations; completeness of follow-up and compliance. The unblinded statistician will prepare these Open Reports.

- *Intended content of material to be available in closed sessions.*
  Closed Reports, available only to those attending the Closed Sessions of the DMC meeting, will include analyses of primary and secondary efficacy endpoints with due allowance for this being a feasibility study; analyses of adverse events and symptom severity; and Open Report analyses that are displayed by intervention group. The unblinded statistician, who is not involved in any decisions relating to the trial, will prepare these Closed Reports for the DMC.

For each DMC meeting, Open and Closed Reports will be provided to DMC members approximately two weeks prior to the date of the meeting by the unblinded trial statistician. The Open and Closed Reports should provide information that is as accurate as possible at the time of preparation, with follow-up that is as complete as possible.

### External evidence

Identification and circulation of published external evidence (e.g. from other trials/ systematic reviews) is a responsibility of the CI. The DMC should continue to be made aware of other data that may impact on the trial.

### Communication

The facilitator will be responsible for the organisation of meetings and should be copied into relevant communications with and between the DMC.

### Confidentiality

DMC members are expected to store securely copies of the DMC reports, agenda and minutes, as well as copies of communications between meetings. All documentation should be considered confidential.

## 8 Decision Making

### TSC / DMC decision-making

To be quorate for decision-making, at least two members (two thirds of the appointed membership) including the Chair will be present. It is important that the implications



Loan JJM, *et al. BMJ Open* 2023; 13:e075187. doi: 10.1136/bmjopen-2023-075187

(e.g. ethical, statistical, practical, and financial) for the trial be considered before any decision is made.

The DMC is jointly responsible with the TSC for safeguarding the interests of participating patients and for the conduct of the trial. Recommendations to amend the protocol or conduct of the study made by the DMC will be considered and accepted or rejected by the TSC. The TSC will be responsible for deciding whether to continue or to stop the trial based on the DMC recommendations.

DMC recommendations include but are not limited to:

- Trial continues as planned
- Early termination of the trial
- Stopping recruitment within a subgroup
- Extending recruitment or extending follow-up (pending approval by the funder)
- Proposing protocol changes

There are no pre-specified stopping rules in this feasibility trial. Should the DMC decide to recommend early termination of the trial, a full vote of the DMC will be required. In the event of a split vote, the decision will go with the majority vote, but a report should be provided to the TSC, written anonymously by the DMC members who are in the minority, for the purposes of officially stating their position on the issue. This report should not include unblinded data unless deemed necessary by the DMC. This information should be forwarded to the trial chief investigator as rapidly as possible.

**Consensus and quoracy**

Every effort should be made to achieve consensus. The role of the Chair is to summarise discussions and encourage consensus; therefore, it is usually best for the Chair to give their own opinion last. If a vote is required to achieve consensus, all independent members of the DMC have the opportunity to cast a vote with the chair voting last. The CI is not able to cast a vote.

## 9 Reporting

**Meeting Minutes**

Two sets of minutes will be prepared: the Open Session Minutes and the Closed Session Minutes.

Minutes of the open session will be prepared by the facilitator on behalf of the CI within two weeks of the meeting, and uploaded to the NIHR MIS when approved. Copies of minutes will be sent to all members, the sponsor and the funder, and a copy will be retained in the Trial Master File. These minutes and actions will be used as a basis for the following DMC meeting agenda.

The method of recording the outcome of the Closed session of the DMC will be at the discretion of the DMC Chair, and will be the responsibility of the DMC members to ensure confidentiality. Minutes of the closed session will be prepared within two weeks of the meeting. Any minutes of record of the Closed session of the DMC

should not be circulated out with the DMC members. Copies will be kept by the DMC chair or other designated DMC member. These will be sent to the trial manager and archived at the time of study closure.

**Recommendations**

Within two weeks of the meeting, the DMC chair/other designated DMC member will report via email to the Trial Manager their recommendations/decisions. The trial manager will forward the DMC meeting report and recommendations to the CI, TSC and the trial management group.

**Disagreements**

If there is a serious disagreement between the DMC and the TSC a meeting of these groups should be held. The information to be shown would depend upon the action proposed and the DMC's concerns. Depending on the reason for the disagreement some confidential data might have to be revealed to all those attending such a meeting. The meeting could be chaired by an external expert who is not directly involved with the trial.

## 10 After the Trial

- Publication of results
- The information about the DMC that will be included in published trial reports
- Whether the DMC will have the opportunity to approve publications, especially with respect to reporting of any DMC recommendation regarding termination of a trial
- Any constraints on DMC members divulging information about their deliberations after the trial has been published

**Publication of results**

DMC members will have the opportunity to read and comment on the proposed main publications of trial data prior to submission and abstracts and presentations during the trial, especially with respect to reporting of any DMC recommendation regarding termination of a trial.

This review may be concurrent to that of the trial investigators and TSC. DMC members will be named and their affiliations listed in the main report, unless they explicitly request otherwise.

**Confidentiality of results**

Unless permission has been agreed with the TSC, individual members will not discuss confidential information to which they have become party as a result of their involvement in the trial until 12 months after the primary trial results have been published.



Loan JJM, *et al. BMJ Open* 2023; 13:e075187. doi: 10.1136/bmjopen-2023-075187

**Appendix 1: Agreement and Competing interests form for DMC members**

*Please complete the following document and return to the DMC Facilitator.*

- ☐ I have read and understood the CARE Trial DMC Charter V2.0
- ☐ I agree to join the Data Monitoring Committee for this trial
- ☐ I agree to treat all sensitive trial data and discussions confidentially

The avoidance of any perception that members of a DMC may be biased in some fashion is important for the credibility of the decisions made by the DMC and for the integrity of the trial. Possible competing interest should be disclosed via the trial office. In many cases simple disclosure up front should be sufficient. Otherwise, the (potential) DMC member should remove the conflict or stop participating in the DMC.

Table 1 lists potential competing interests.

- ☐ **No,** I have no competing interests to declare
- ☐ **Yes,** I have competing interests to declare (please detail below)

Please provide details of any competing interests:

| |
|---|
| |

Name: ___________________________

Signature: ___________________________ Date: ______________

**Table 1**

- Stock ownership in any commercial companies involved
- Stock transaction in any commercial company involved (if previously holding stock)
- Consulting arrangements with the sponsor
- Frequent speaking engagements on behalf of the intervention
- Career tied up in a product or technique assessed by trial
- Hands-on participation in the trial
- Involvement in the running of the trial
- Emotional involvement in the trial
- Intellectual conflict eg strong prior belief in the trial’s experimental arm
- Involvement in regulatory issues relevant to the trial procedures
- Investment (financial or intellectual) in competing products
- Involvement in the publication



Loan JJM, *et al. BMJ Open* 2023; 13:e075187. doi: 10.1136/bmjopen-2023-075187
